# Effect of Anionic Lipids on Mammalian Plasma Cell Membrane Properties

**DOI:** 10.1021/acs.langmuir.2c03161

**Published:** 2023-02-09

**Authors:** Alexandra L. Martin, Philip N. Jemmett, Thomas Howitt, Mary H. Wood, Andrew W. Burley, Liam R. Cox, Timothy R. Dafforn, Rebecca J. L. Welbourn, Mario Campana, Maximilian W. A. Skoda, Joseph J. Thompson, Hadeel Hussain, Jonathan L. Rawle, Francesco Carlà, Christopher L. Nicklin, Thomas Arnold, Sarah L. Horswell

**Affiliations:** ^†^School of Chemistry and ^‡^School of Biosciences, University of Birmingham, Edgbaston, BirminghamB15 2TT, U.K.; § ISIS Pulsed Neutron and Muon Source, Science and Technology Facilities Council, Rutherford Appleton Laboratory, Harwell, OxfordshireOX11 0QX, U.K.; ∥ Diamond Light Source, Harwell Science and Innovation Campus, Chilton, Didcot, OxfordshireOX11 0DE, U.K.; ⊥ European Spallation Source ERIC PO Box 176, SE-221 00Lund, Sweden; # Department of Chemistry, University of Bath, Claverton Down, BathBA2 7AY, U.K.

## Abstract

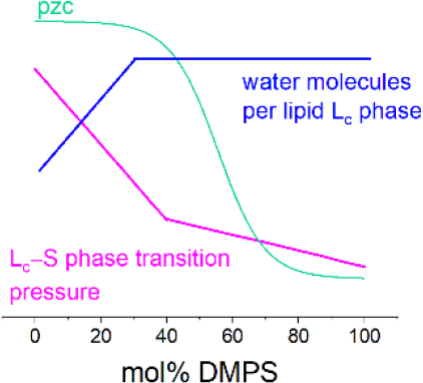

The effect of lipid composition on models of the inner leaflet of mammalian cell membranes has been investigated. Grazing incidence X-ray diffraction and X-ray and neutron reflectivity have been used to characterize lipid packing and solvation, while electrochemical and infrared spectroscopic methods have been employed to probe phase behavior in an applied electric field. Introducing a small quantity of the anionic lipid dimyristoylphosphatidylserine (DMPS) into bilayers of zwitterionic dimyristoylphosphatidylethanolamine (DMPE) results in a significant change in the bilayer response to an applied field: the tilt of the hydrocarbon chains increases before returning to the original tilt angle on detachment of the bilayer. Equimolar mixtures, with slightly closer chain packing, exhibit a similar but weaker response. The latter also tend to incorporate more solvent during this electrochemical phase transition, at levels similar to those of pure DMPS. Reflectivity measurements reveal greater solvation of lipid layers for DMPS > 30 mol %, matching the greater propensity for DMPS-rich bilayers to incorporate water. Taken together, the data indicate that the range of 10–35 mol % DMPS provides optimum bilayer properties (in flexibility and function as a barrier), which may explain why the DMPS content of cell membranes tends to be found within this range.

## 1 Introduction

Biological cell membranes are key to the function of the cell, forming a selective barrier between the cell and its environment or between the cytosol and the aqueous fluid phases of different compartments within the cell.^1^ They are formed of bilayers of lipid molecules, in which are contained various functional molecules, receptors, and proteins.^1^ While there has been longstanding interest in membrane proteins, the importance of the lipid constituents has more recently begun to attract interest,^2−4^ in part because the action of the proteins is influenced by the local mechanical properties (fluidity) of the membrane,^5−7^ the charge of the lipid headgroups, and specific interactions with the headgroups.^8−10^ For example, proteins may be preferentially located in less-fluid or more-fluid domains^11,12^ or may be surrounded by an annulus of a particular lipid type.^11^ Lipids of various types are also essential in cell-signaling processes.^8,13,14^ To understand fully the role of lipids in membrane function, it is necessary to build a picture of how the lipids’ molecular structures affect the properties of the membranes they form. For this undertaking, it is common to use model systems where the composition and environment can be more precisely controlled,^15−17^ such as vesicles, monolayers, and supported bilayers.^16,17^ A range of structural probes may then be employed, including X-ray^18−24^ and neutron^25−29^ scattering techniques, vibrational spectroscopies,^30−32^ and imaging methods,^33−36^ inter alia. One of the fundamental questions remaining to be answered is the reason for the structural diversity of lipids in nature. Experiments investigating the effect of composition on lipid ensemble properties can shed light on the roles of different lipid types, on the properties of different membrane types, and lipid interactions with other functional molecules.

**Figure 1:**
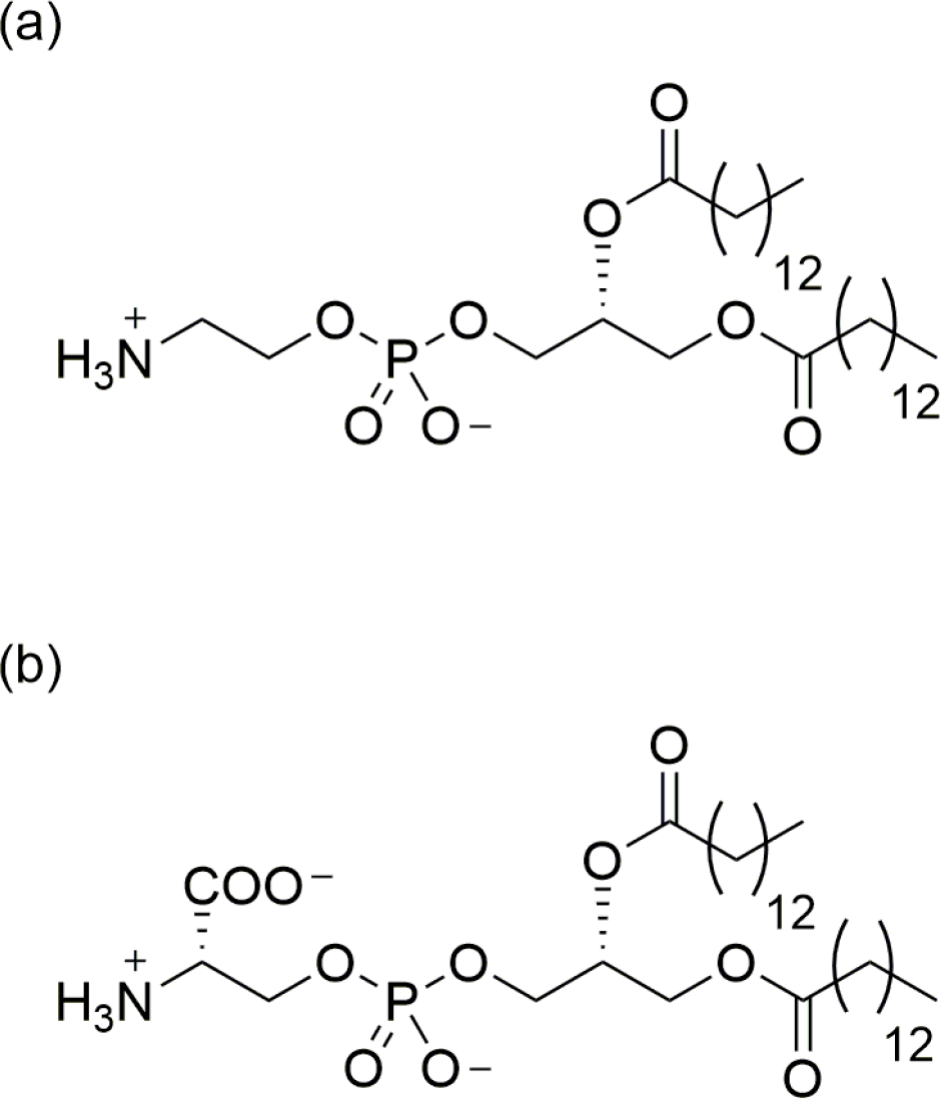
Structures of (a) dimyristoylphosphatidylethanolamine (DMPE) and (b) dimyristoylphosphatidylserine (DMPS).

As one of the primary functions of a cell membrane is to provide a barrier to the passage of ions and water molecules,^1^ electrochemical experiments are employed to investigate the barrier properties of membranes, using patch clamp methods,^37^ or of their mimics, using an array of electrochemical tools to study suspended,^38−40^ supported,^41−45^ tethered,^46,47^ or floating^48^ monolayers or bilayers. The application of an electrical potential difference across a bilayer also mimics the effects of ion gradients or the charge asymmetry that arises from the asymmetric distribution of charged lipids over the two halves of a bilayer.^45^ A continuously tunable electric field of similar magnitude to those found in nature can be generated easily,^45,49^ and if the bilayer is supported on or in proximity to a flat surface, then in situ vibrational spectroscopy,^50−55^ neutron reflectivity,^49,55,56^ and scanning probe microscopies^57−60^ can be used to examine the effect of the field on the lipid organization and bilayer structure. This strategy has been adopted to investigate the phase behavior of lipid layers under the influence of an applied field,^49−51,56,57,59,60^ the effect of molecular structure on the phase behavior,^52,53^ and the interaction of small peptides with bilayers of various compositions.^48,52,58^ Two common lipid types in mammalian cells, phosphatidylethanolamine (PE) and phosphatidylserine (PS) (Figure 1), have received relatively little attention compared with the phosphocholines (PC) and signaling lipids. PE and PS have smaller headgroups than PC, which may explain in part their abundance on the inner (cytosolic) leaflet of the mammalian plasma cell membrane.^1^ PS has an anionic headgroup and is found almost exclusively on that side;^1,2^ its presence in the outer (exoplasmic) side of the membrane induces a process leading to cell death. Some structural studies of PE^18,21,61^ and PS^62,63^ lipids and a few of PC/PS mixtures^64^ have been reported, but there are few on PE/PS mixtures.^56^ PE/PS mixtures are an interesting case because the similarity in molecular geometry and headgroup structure might be expected to result in ideal mixing of the two lipid types (both headgroups may participate in interheadgroup hydrogen bonding), although PS is anionic while PE is zwitterionic, which may affect the distribution of PS and PE within the layer. That PE and PS lipids are both predominantly found in the inner leaflet of the mammalian cell membrane suggests that benefit lies in the study of the mixtures, but comparatively few such studies exist.^56,65^ There are none, to our knowledge, exploring the effect of varying the anionic lipid content.

In this work, we investigate the effect of composition on the structure and properties of mixtures of dimyristoylphosphatidylethanolamine (DMPE) and dimyristoylphosphatidylserine (DMPS). These two lipids are structurally very similar (Figure 1) and have similar sizes and shapes but behave differently in an applied electric field: DMPE bilayers are tightly packed with low solvent content and do not show noticeable structural changes upon application of an electric field,^53^ whereas DMPS bilayers respond with an increase in the chain tilt angle and solvent content at negatively charged surfaces.^54^ In this study, we show that mixing of the lipids leads to intermediate bilayer properties and a different structural response to the applied field. To shed light on the differences between mixtures and their two components, we use a combination of grazing incidence X-ray diffraction (GIXD), X-ray reflectivity (XRR), and neutron reflectivity (NR) to characterize the structure of the monolayers from which our bilayers are formed, over a range of lipid phases. The addition of DMPS to a DMPE monolayer results in the formation of a solid phase over a wider range of molecular area. Subtle differences in packing and in the tendency for monolayers at intermediate molecular area to take up solvent are observed for some mixtures, which may explain the different responses and take-up of solvent observed for mixed bilayers on increasing the applied electric field.

## 2 Experimental Section

### 2.1 Materials

Dimyristoylphosphatidylethanolamine (DMPE), dimyristoylphosphatidylserine sodium salt (DMPS), and their perdeuterated analogues (D54-DMPE, D54-DMPS) were purchased from Avanti Polar Lipids (Birmingham, AL) and used as received. Solutions were prepared in a 9:1 v/v ratio mixture of chloroform and methanol (both HPLC grade, Sigma-Aldrich).

Ultrapure water was used throughout. A tandem Elix-Milli-Q A10 system (Millipore, France) was used at Birmingham, a Millipore system was used at ISIS, and a Purelab Classic UV system (Elga, U.K.) was used at Diamond Light Source. Deuterium oxide (99.9% D) obtained from Sigma-Aldrich was employed for spectroelectrochemical and neutron measurements. Neutron reflectivity measurements were carried out with a subphase of D_2_O or of air-contrast-matched water (ACMW, prepared as 8% v/v D_2_O in H_2_O). Electrolyte solutions were prepared from sodium fluoride (Puratronic [99.995% metals basis], Alfa Aesar, U.K.) at a concentration of 0.1 M in ultrapure water or D_2_O.

Volumetric glassware was cleaned with piranha solution (***Caution!**This is a highly exothermic process that may cause an explosion!*), followed by rinsing thoroughly with ultrapure water, soaking overnight in ultrapure water, and further rinsing with ultrapure water before use. All other glassware was cleaned by heating in a 1:1 mixture of nitric and sulfuric acids for ∼1 h, followed by thorough rinsing and soaking overnight in ultrapure water. PTFE and Kel-F parts of the spectroelectrochemical cell were cleaned with a 1:1 mixture of 30% ammonia solution and 30% hydrogen peroxide solution and then thoroughly rinsed with ultrapure water, soaked overnight in ultrapure water, rinsed again, and dried in a designated clean oven.

### 2.2 Langmuir Trough Measurements

A Nima Langmuir trough equipped with a Delrin barrier and a dipping mechanism was used to record isotherms and to deposit bilayers on electrodes. A thermostatted water bath was used to control the subphase temperature. The temperature used for isotherm measurements was 19 °C, below the gel–liquid crystalline phase-transition temperature of either lipid (DMPE: 50.4 °C in ref (66), 49 °C in ref (67); DMPS: 39 °C^68^). The trough was cleaned with chloroform before being filled with ultrapure water and allowed to reach thermal equilibrium. The surface was checked for cleanliness by ensuring the pressure did not rise as the area was reduced. Next, a fixed volume (typically 80 μL) of a chloroform/methanol solution of lipid was added to the water surface. The solvent was allowed to evaporate, and isotherms were recorded. To create lipid bilayers, a freshly cleaned Au(111) crystal (sections 2.4 and 2.5) was immersed in the subphase before deposition of lipids onto the water surface. An isotherm was recorded, and the monolayer was then compressed to a target pressure of 47 mN m^–1^ (to match the pressure used in previous reports for the pure lipids^53,54^). The crystal was withdrawn vertically through the interface at a rate of 2 mm min^–1^ (Langmuir–Blodgett deposition), dried for 30 min in argon, and then lowered onto the water surface at the same dipping rate in a Langmuir–Schaefer configuration (horizontal touch) to deposit the second monolayer and so form a Y-type bilayer. (Au(111) is used for electrochemical structural measurements because it is a relatively flat surface with a wide potential window that facilitates the investigation of structure over a wide range of the applied electric field. Although it is less hydrophilic than quartz (often used in reflectivity measurements), it is sufficiently hydrophilic to allow the formation of Y-type bilayers.) The transfer ratio was 1, and bilayers were stable on the Au surface on the time scale of the electrochemical and IR measurements.

### 2.3 Brewster Angle Microscopy Measurements

Brewster angle microscopy (BAM) was carried out at Diamond Light Source with a Nanofilm EP3SE Imaging Ellipsometer (Accurion), equipped with a 50 mW laser emitting light at 532 nm, a 50× magnification objective, a polarizer, an analyzer, and a CCD camera. p-Polarized light was used, incident upon the water surface of a Langmuir trough (Nima) at the Brewster angle for the air|water interface (53.15°). Images were recorded at a barrier compression speed of 45 cm^2^ min^–1^ (total trough area ca. 700 cm^2^), and those presented herein were acquired on the second compression of each monolayer, for consistency with the X-ray and neutron measurements. The images shown were acquired at the same fixed molecular areas for each composition, one chosen to be within the midplateau region of the isotherm at each composition and the other at the highest molecular area used for X-ray and neutron measurements (section 2.6).

### 2.4 Electrochemical Measurements

Electrochemical measurements were performed in an all-glass three-electrode cell, with a Au(111) single crystal, oriented to better than 0.5° (MaTecK GmbH, Jülich, Germany), as a working electrode, a Au coil (99.999%, Alfa Aesar, U.K.) as a counter electrode, and a saturated calomel electrode (Hach Lange) as a reference electrode. The reference electrode was immersed in saturated potassium chloride solution and connected to the rest of the cell via a salt bridge. Although the saturated calomel electrode was used for electrochemical measurements, a Ag|AgCl|3 M KCl electrode was used for infrared measurements and so potentials in this work will be reported vs Ag|AgCl|3 M KCl. The Au(111) crystal was cleaned using a method previously described in the literature:^69^ the crystal was flame-annealed and allowed to cool to ambient temperature; a drop of ultrapure water was then placed on the surface. The crystal was then heated gently to remove the drop, which was immediately replaced with a fresh drop. The crystal was then transferred to the cell or Langmuir trough with the drop of ultrapure water to protect the surface from contamination. NaF (0.1 M) was used as the electrolyte and was purged of oxygen by bubbling with argon for at least 45 min before measurements. An argon blanket was maintained over the solution for the duration of the measurements.

The potentiostat used was a Heka PGStat 590 (Heka, Germany), controlled with a PC with in-house-written software (kindly provided by Dr A. L. N. Pinheiro, Universidade Tecnologica Federal do Parana, Londrina, Brazil) and a data acquisition board (M-series, National Instruments). Chronocoulometry measurements consisted of applying a series of potential steps and recording the resulting current transients, as described previously.^51,53^ The potential was held at a base potential of −0.06 V, stepped to the potential of interest and held for 3 min (to allow adsorption equilibrium to be reached), and then stepped to a potential at which desorption takes place before being returned to the base potential. The current transients were recorded during the desorption step. The sequence was recorded for potentials every 0.05 V, starting from 0.44 V and moving in the cathodic direction. The current transients were integrated to give the total charge passed during each potential step. The resulting relative charge densities were then converted to absolute charge densities using the potential of zero charge (pzc) of the bare electrode (0.315 V).

### 2.5 Spectroelectrochemical Measurements

A custom-built spectroelectrochemical cell was used for polarization-modulated infrared reflection absorption spectroscopy (PM-IRRAS) measurements. The working electrode was a Au(111) crystal, oriented to better than 0.5° (MaTecK GmbH, Jülich, Germany), and was prepared as described in section 2.4. The counter electrode was a gold coil (99.995%, Alfa Aesar), arranged concentric to the working electrode, and the reference electrode was a Ag|AgCl|3 M KCl electrode (BASi, U.S.). The window was a 1 in. barium fluoride equilateral prism (Crystran, U.K.) and was cleaned before use with methanol, then with water and then in an ozone chamber. The electrolyte used was 0.1 M NaF in D_2_O. Spectra were recorded at a temperature of 19 °C (±1 °C), where both lipids are in the gel phase.^66−68^

Measurements were carried out with a Bruker Vertex 80v spectrometer equipped with a PMA50 module, with the latter comprising a photoelastic modulator with a ZnSe 50 kHz optical head (PEM-100, Hinds Instruments) and a synchronous sampling demodulator (GWC Technologies) to obtain the difference signal. The half-wave retardation was set to 2900 cm^–1^ for investigating the C–H stretching region of the spectrum and to 1600 cm^–1^ for the C=O stretching region. Spectra were acquired at an instrumental resolution of 2 cm^–1^. The angle of incidence was 51° for the C–H stretching region and 61° for the C=O stretching region; the optimum thicknesses of electrolyte between the working electrode and the window were taken as 1.9 and 3.6 μm, respectively.^70^ Fresnel 1 software,^71^ kindly provided by Dr V. Zamlynny (Acadia University, Canada), was employed to determine the electrolyte thickness and to simulate theoretical spectra of randomly oriented molecules for the cell configurations used in the experiments. These simulated spectra are used in the analysis of PM-IRRA spectra, as described by Zamlynny and Lipkowski.^72^ The isotropic optical constants required for these simulations were as acquired previously;^53,54^ weighted averages were calculated for the mixtures from the optical constants of the pure components. Spectra were demodulated and corrected for the response of the photoelastic modulator as described in ref (72).

### 2.6 Reflectivity and Diffraction Measurements

Neutron reflectivity measurements were carried out at the INTER^73^ and SURF^74^ beamlines at ISIS Pulsed Neutron and Muon Source (Oxfordshire, U.K.). Data were acquired in time-of-flight mode over two angles (0.8 and 2.3°) at INTER and three angles (0.35, 0.7, and 1.5°) at SURF at a resolution of 7%; both beamlines employ ^3^He detectors. In each case, a large-area (700 cm^2^), temperature-controlled Langmuir trough (Nima) was used and encased in a box to reduce the exchange of D_2_O with atmospheric water. The in-house written software Mantid^75,76^ was used for data reduction.

X-ray reflectivity and grazing incidence X-ray diffraction measurements were carried out on the I07 beamline at Diamond Light Source (Oxfordshire, U.K.).^77^ X-rays (12.5 keV, λ = 0.9919 Å) were directed onto the water surface with a double-crystal deflector system.^78^ A large-area (700 cm^2^) Langmuir trough (Nima) with temperature control was employed and encased in a box with a He atmosphere to reduce background scattering and beam damage. XRR data were collected over a *q*_*z*_ range of 0.018 to ∼0.6 Å^–1^, where *q*_*z*_ is momentum transfer normal to the surface, and were reduced with an in-house Python script. GIXD was measured with an angle of incidence corresponding to *q*_*z*_ = 0.018 Å^–1^ and with a pinhole setup,^23^ allowing the acquisition of diffraction images. These were subsequently scaled and spliced to produce an image over a *q*_*z*_ range of 0–0.8 Å^–1^, using an in-house-written MATLAB script. Reflectivity data were fitted using RasCal.^79^ GIXD data were integrated and fitted using MATLAB scripts and Origin Pro.

X-ray measurements were carried out at two fixed pressures and at four fixed molecular areas. The fixed pressures were both in the solid phase, 47 mN m^–1^, to match the deposition pressure used for the bilayers, and 40 mN m^–1^, which is comfortably in the solid phase for each composition and often used for the deposition of bilayers in other electrochemical structural measurements.^45^ The fixed molecular areas were chosen to span the remaining condensed-phase region of the isotherm: 38, 42, 44, and 46 Å^2^ on the isotherm, hereafter referred to as areas A1, A2, A3, and A4, respectively. Neutron measurements were carried out for a subset of these points at 40 mN m^–1^ and at the highest and lowest fixed areas (A1 and A4). Two contrasts were employed: lipids with perdeuterated chains (d-lipids) on D_2_O and d-lipids on air-contrast-matched water (ACMW). X-ray measurements employed undeuterated lipids (h-lipids) for all compositions, but measurements were also carried out with d-lipids for the compositions and pressures used for NR measurements to ensure that the structures were comparable.

## 3 Results and Discussion

### 3.1 Isotherms

**Figure 2:**
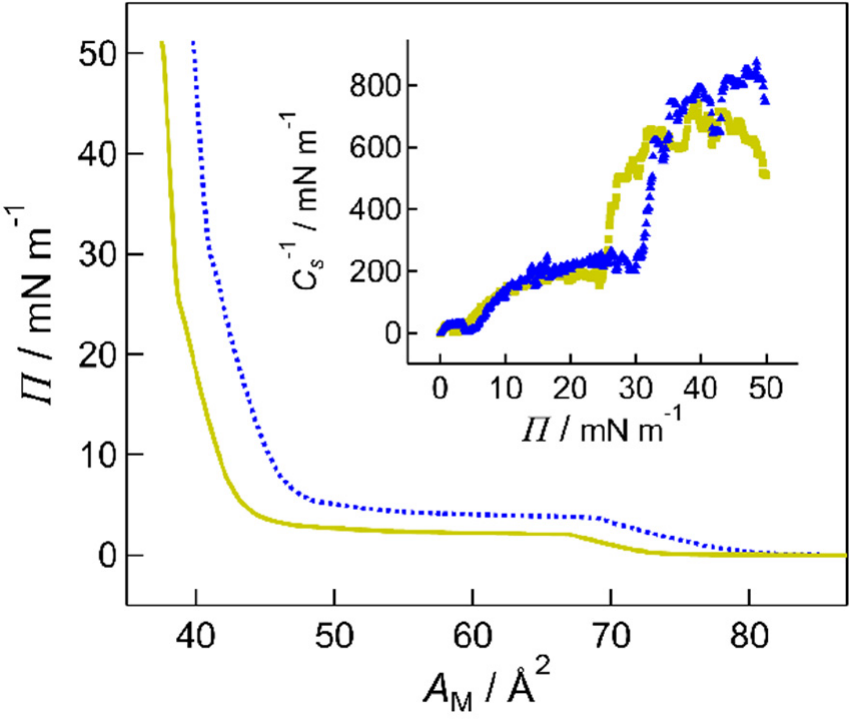
Isotherms of 9:1 (blue dashed line) and 1:1 (yellow solid line) mixtures of DMPE and DMPS. Inset: Compressibility moduli derived from the isotherm data, plotted as a function of molecular area, with blue triangles representing the 9:1 mixture and yellow squares representing the 1:1 mixture.

Figure 2 presents isotherms of mixtures of DMPE and DMPS in ratios of 9:1 and 1:1 PE:PS. Isotherms of DMPE, DMPS, and the remaining mixtures are provided in Figure S1 of the Supporting Information. The DMPE and DMPS isotherms are consistent with those in our previous publications.^53,54^ The surface pressure of the phase transition between the liquid expanded (L_e_) and liquid condensed (L_c_) phases (the plateau in the isotherm) decreases as the proportion of DMPS is increased. Likewise, the pressure at which the L_c_ phase condenses into the solid phase (the kink in the isotherm) also decreases with increasing DMPS content (Figure S2a). The limiting areas per molecule (obtained by extrapolating the solid-phase portion of the isotherm to the abscissa) are all similar at around 40 Å^2^. The gradient of the isotherm at a given point is related to the compressibility of the monolayer: the steeper the gradient, the less compressible the monolayer. The concept is quantified through differentiation of the isotherm and calculation of the compressibility modulus, *C*_*s*_^–1^, from eq 1(80)
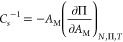
1where Π is the surface pressure, *A*_M_ is the area per molecule, *N* is the number of molecules, and *T* is the absolute temperature. *C*_*s*_^–1^ is plotted as a function of surface pressure in the inset to Figure 2. The maximum values of *C*_*s*_^–1^ are around 700–800 mN m^–1^ and vary little with composition, which indicates that the monolayers have similar mechanical strength.

Calorimetry results reported in the literature for aqueous dispersions suggest that the mixing of DMPE and DMPS is close to ideal,^81^ presumably because the molecules are of similar size and shape and their intermolecular interactions have similar natures. An indication of the mixing behavior in planar layers can be obtained by considering the excess area and excess Gibbs energy of mixtures. The excess area *A*^exc^ is the difference in the measured area per molecule of the mixture (*A*_12_) and the weighted-average molecular areas of the constituent molecules (*A*_1_ and *A*_2_)^82^
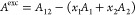
2where *x*_*i*_ is the mole fraction of species *i*. At all pressures, *A*^exc^ of each mixture is small and of similar magnitude to the expected experimental error. Most mixtures have slightly positive *A*^exc^, but the magnitude of the values suggests that mixing is close to ideal for these lipids.

The excess Gibbs energy of a mixture was calculated from eq 3(80,83)
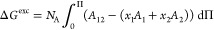
3and is plotted as a function of monolayer composition in Figure S2 for the monolayers at a surface pressure of 47 mN m^–1^. If it is assumed that the excess Gibbs energy is related to differences in intermolecular interactions between molecules in mixed or segregated films, then the data show that the mixing of DMPE and DMPS is close to but not quite ideal. At most compositions, the excess Gibbs energy is positive, an indication that the DMPE–DMPS interactions are less favorable than in the ideal mixture, the weighted sum of the DMPE–DMPE and DMPS–DMPS interactions. This may be a result of the disruption of an organized hydrogen bonding network by accommodating slightly different headgroup geometries. Both DMPE and DMPS have strong dispersion interactions between their chains and hydrogen bonding interactions between their headgroups. Hydrogen bonding interactions are directional and can result in particular headgroup conformations that may differ for DMPE and DMPS (the latter has two hydrogen bond acceptors and so more possible arrangements). Introduction of DMPS molecules into DMPE monolayers (or vice versa) may disrupt the headgroup organization to some extent, resulting in a slightly positive excess area and excess Gibbs energy without preventing mixing (i.e., the interactions between like molecules are generally stronger than those between unlike molecules). If the excess Gibbs energy is added to the Gibbs energy of an ideal mixture, *RT*(*x*_1_ ln *x*_1_ + *x*_2_ ln *x*_2_), to obtain the total Gibbs energy of mixing, then the values are all negative, which indicates that the two components still mix. Mixing is most favorable at the 1:1 ratio (with a very slightly negative excess energy), which may indicate that molecules can adopt an optimum arrangement of headgroups in this composition. The observation that the excess Gibbs energy tends to be more positive for mixtures of low DMPS content than for those of high DMPS content suggests that the disruption of the network of DMPE headgroups is more unfavorable than the disruption of the packing of DMPS headgroups or that the latter is offset to some extent by the dilution of charged species within the monolayer. Wydro studied the mixing of cholesterol with monolayers formed of dipalmitoyl PE and PS on different subphases.^65^ On water, the binary mixture of *x*_PS_/*x*_PE_ = 0.53 exhibited negative excess Gibbs energy at 32.5 mN m^–1^ (also in the solid phase), which was attributed to the ability of both molecules to form intermolecular hydrogen bonds.^65^

### 3.2 Electrochemical Measurements

**Figure 3:**
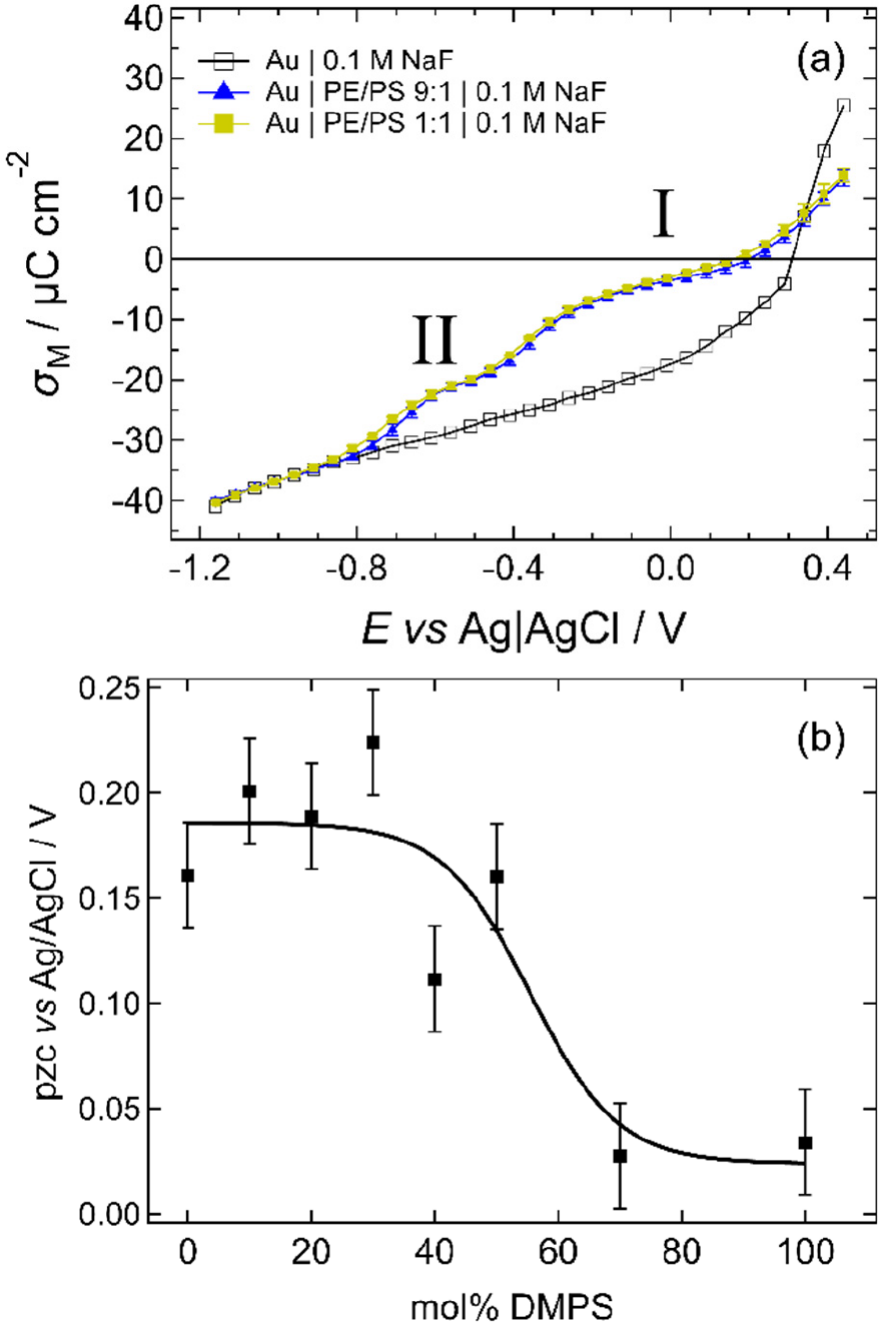
(a) Charge density–potential plots for uncoated Au(111) (open squares) and Au(111) with bilayers of DMPE:DMPS mixtures 9:1 (filled triangles) and 1:1 (filled squares). (b) pzc plotted as a function of mol % DMPS. The error bars have a length of 50 mV (corresponding to the potential step size), and the line is a guide for the eye.

Figure 3a shows chronocoulometry data acquired for the 9:1 and 1:1 DMPE/DMPS mixtures. The data for other compositions are given in Figure S3. The mixtures have similar electrochemical phase behavior to one another and to that reported in previous publications.^45,49−51,53,54,56^ The plots measured for surfaces coated in lipid bilayers show two steps: the first (into region II) arises from the adsorption/desorption of the lipid bilayer and the second (between regions II and I) is from a phase change between two different structures of the bilayer.^45,49^ In situ NR studies for the case of DMPC/cholesterol bilayers have shown that in the potential range positive of −0.4 V (region I) the bilayer is directly adsorbed on the Au(111) surface and in the range between −0.4 V and desorption (region II) the bilayer is separated from the electrode surface by a cushion of electrolyte. The step in charge density is related to the movement of electrolyte through the bilayer to form this cushion.^45,49^ For the adsorbed layer, the slope of the charge density–potential plot gives the capacitance of the interface, which is lower in the presence of lipid than in the absence of lipid because the planes of charge (the metal and the outer Helmholtz plane) are farther separated and the average permittivity of the organic layer is lower than that of interfacial water. The data for the PE/PS mixtures show that these slopes are similar for the different lipid compositions up to 50% DMPS but are higher for 70% DMPS and pure DMPS. The higher interfacial capacitance for DMPS has previously been attributed to higher solvent content within the bilayer, which increases the average permittivity of the bilayer.^54^ Therefore, the data in Figure 3 and Figure S3 suggest that the bilayer solvent content changes little with composition between 10 and 50% DMPS.

The chronocoulometry data show that the lipids are adsorbed within a range of charge density of ca. −10 μC cm^–2^ and +15 μC cm^–2^ (region I), which is typical for phospholipid bilayers supported on Au(111) surfaces.^45,49−51,53,54,56^ This range of charge density corresponds to the range of the electric field within which natural cell membranes are stable.^45,49^ The potential of zero charge (pzc) is shifted in the presence of molecules to more negative potentials, as has been observed previously for supported lipid bilayers. The shift indicates a small charge asymmetry across the bilayer.^51^ For zwitterionic molecules, this has been interpreted as a different orientation of the headgroup dipoles in each leaflet of the bilayer. For the anionic molecules, a much larger shift would be expected.^84^ The small size of the shift indicates that the molecules are probably coadsorbed with the counterions.^54,85^ The shift in pzc is largest for DMPS. The mixtures with DMPS content <30% exhibit a similar shift to DMPE, and the 70% mixture has a similar shift to DMPS. The 40 and 50% DMPS mixtures have intermediate shifts. Bearing in mind that the step size in these experiments is 50 mV, small differences in pzc should not be overinterpreted. However, it is possible to comment from these data that at low DMPS concentrations within the bilayer the surface potential is not strongly dependent on composition and that differences emerge at 40% DMPS and above (Figure 3b). Moncelli et al. suggested for lipid monolayers on a mercury electrode that while a PC headgroup was near-planar, the PS headgroup could as well be oriented with its phosphate group closer to the chain portion of the monolayer as with all three charges within the plane.^86^ Becucci et al. also pointed out that there is a contribution to the overall dipole from the orientation of water molecules associated with the ester carbonyl groups within the headgroup portion of the monolayer.^87^ For mixtures, it is possible that the headgroup packing and orientation change slightly as the concentration of anionic lipid exceeds a certain value, for example, if charged groups (such as phosphate) move deeper into the bilayer, as suggested by Moncelli et al.,^86^ or if ester groups have different orientations. It should also be noted that there is a possibility that the distribution of the anionic lipid across the two halves of the bilayer changes with time, as “flip-flop” of lipids between two halves of a supported bilayer has been observed previously with sum frequency vibrational spectroscopy^88,89^ and NR.^90^ Such a change in lipid distribution would alter the charge distribution across the bilayer and so affect the pzc. However, our previous electrochemical study of asymmetric bilayers of DMPE and DMPS showed that there was consistently less negative charge density when the bilayer was prepared with DMPS on the Au-facing side of the interface than when it was prepared with DMPS on the solution-facing side or with the lipids mixed in both sides (equivalent to our 1:1 mixture in the present work).^85^ That the curves did not merge as the experiment proceeded indicates that the rate of flip-flop is slow on the time scale of these measurements (∼3 h), at least for these lipids. The slow rate of flip-flop was attributed to tight packing of the lipids within the bilayer, which raises the activation barrier for traversing the membrane. The flip-flop of lipids observed with NR was shown to take place only for lipids in their fluid phases,^90^ and in other cases, bilayer asymmetry could be maintained over many hours.^90,91^ In addition, previous in situ IR studies of bilayers on Au(111), where one-half of the bilayer is deuterated and the other not,^92,93^ have shown that tilt angles of the undeuterated chains were different for the Au-facing and solution-facing monolayers across the whole range of potentials studied. Given that the IR measurement is longer in duration than the electrochemistry measurement, this would also suggest that the lipids are not redistributing over the time scale in the electrochemical measurements reported here. Mixing DMPS with DMPC in different ways also resulted in subtle differences in the electrochemical response, while the asymmetric bilayers formed from DMPE and DMPC (both zwitterionic) were very similar in their responses.^85^ These previous reports thus indicate that the changes in pzc with anionic lipid content are more likely to arise from differences in how DMPS packs within a mixed monolayer than from any change in the distribution of anionic lipid across the bilayer.

### 3.3 Spectroelectrochemical Measurements

#### 3.3.1 C–H Stretching

**Figure 4:**
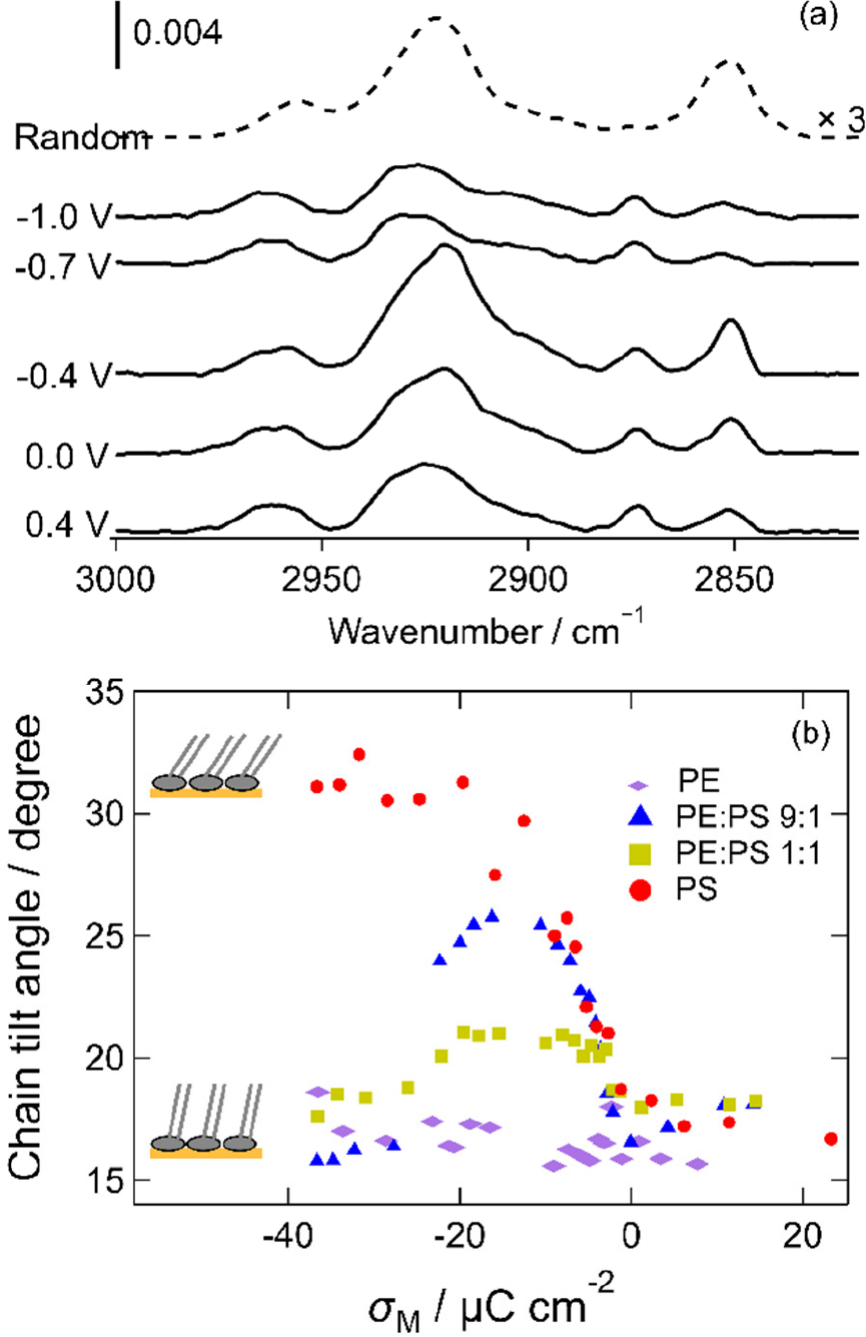
(a) Selected IR spectra of the 9:1 mixture in the C–H stretching region at the indicated potentials. The dashed line is the calculated spectrum for randomly oriented molecules. (b) Plot of the tilt angle of the hydrocarbon chain backbone from the surface normal as a function of charge. Data for DMPE and DMPS are reproduced from refs (53) and (54), respectively, with permission. Copyright 2013 and 2014, the authors under a Creative Commons (CC-BY) license. Error bars omitted for clarity (±3°).

Figure 4a shows a series of in situ infrared spectra acquired in the C–H stretching region for the 9:1 mixture. Spectra for the 1:1 mixture are given in the Supporting Information (Figure S4). The methyl groups on the ends of the hydrocarbon chains have two vibrational modes in this region: the symmetric stretching mode at ∼2870 cm^–1^ and the asymmetric stretching mode at ∼2960 cm^–1^.^66,67,94−96^ The methylene CH_2_ groups have a symmetric stretching mode, υ_s_, at ∼2850 cm^–1^ and an asymmetric stretching mode, υ_as_, at ∼2920 cm^–1^.^66,67,94−96^ There are also two Fermi resonances in this region of the spectrum, which result from the combination of the CH_2_ bending mode overtone with the symmetric stretching mode.^96,97^ The spectra were fitted to six peaks, using a mixed Gaussian–Lorentzian line shape, as described previously.^53^ An example is given in the Supporting Information, Figure S5. The peak positions provide information on the degree of ordering in the hydrocarbon chains: a higher wavenumber is associated with a larger number of *gauche* conformers in the chains.^66,67,94,95^ The peak positions for the 1:1 mixture vary little across the potential range. The average positions for the symmetric and asymmetric stretching modes are 2851.6 (±0.4) and 2920.5 (±0.4) cm^–1^, respectively. There is more variation in the symmetric stretching mode for the 9:1 mixture, with a slight increase in wavenumber for the desorbed bilayer, but the change is within error. The average positions are 2851.6 (±0.9) and 2919.9 (±0.4) cm^–1^. The symmetric stretching mode in the mixtures is similar to that previously observed for DMPE bilayers and 0.5–1.0 cm^–1^ higher than for DMPS bilayers; the position of the asymmetric stretching mode is around 1.0–1.5 cm^–1^ higher than for DMPE and DMPS bilayers. For comparison, DMPC stretching modes are at higher wavenumbers (2852.5–2854.0 and 2922.0–2923.0 cm^–1^) because DMPC molecules pack less tightly than DMPE or DMPS. The results indicate that lipid molecules in mixtures of DMPE and DMPS have relatively few *gauche* conformers and are in the gel state,^66,94,95^ as are the pure components and as would be expected at this temperature. The peak width gives an indication of the degree of mobility of the molecules. For the 1:1 mixture, the full widths at half-maximum (fwhm) are 9.8 (±0.8) and 16.3 (±1.0) cm^–1^ for the symmetric and asymmetric stretching modes, respectively, similar to the values reported for DMPE at negative potentials.^53^ The fwhm values in the spectra of the 9:1 mixture show a small increase when the bilayer is detached, suggesting a slightly greater degree of mobility of the molecules in this state, but the values for the adsorbed bilayer (9.1 and 18.9 cm^–1^) are similar to those of the 1:1 mixture and to DMPE at positive potentials. Apparently, the mixing of the molecules does not result in a significant change in chain organization or mobility for the PE/PS mixtures. PS is often studied in the presence of different metal ions, particularly calcium because of its importance in physiological processes such as membrane fusion and signal transduction.^98^ Calcium has an ordering effect on PS lipids relative to the ammonium or sodium salts, binding to the phosphate group by replacing a water molecule and causing crystallization of the lipid.^98^ The effect of this ordering on the C–H stretching region of the spectrum is mainly to increase the phase-transition temperature, with a slight lowering of the CH_2_ symmetric stretching band position in the gel phase (∼1 cm^–1^).^98^ A study of the effect of calcium on our bilayers is complicated by the specific adsorption of the counterions on the electrode surface, so the present study was restricted to sodium-containing electrolyte.

Finally, the integrated areas of the peaks can be used to determine the orientation of the associated transition dipole moments and, from these, the chain tilt angle with respect to the surface normal (Supporting Information).^50,72^ This chain tilt angle is plotted in Figure 4b as a function of charge density for the 9:1 and 1:1 mixtures. Data for DMPE^53^ and DMPS^54^ are included for comparison. Although the applied potential is the directly controlled variable, the resulting charge density is the driving force for the observed changes in molecular orientation and solvent distribution at the interface. As the charge on the metal becomes negative, the tilt angle of the chains increases and then falls to its original value when the molecules are detached from the surface. The response is seen for both mixtures but is much stronger for the 9:1 mixture. The behavior differs from those of the constituent molecules: DMPE exhibits almost no response to the applied field,^53^ whereas the tilt angle of DMPS increases at negative charge densities and remains high when the molecules are detached.^54^ However, the behavior is similar to that observed for DPPC bilayers^99^ and asymmetric DMPE bilayers where one of the monolayers is deuterated.^93^ The negligible response of the DMPE bilayer to the applied field has previously been attributed to a combination of tight packing between molecules and strong hydrogen bonding interactions between the headgroups. DMPS bilayers differ in that the anionic headgroup requires stronger solvation at negatively charged surfaces. It is likely that the addition of a small quantity of DMPS into the DMPE bilayer disrupts the packing and allows the bilayer to respond to the field or that the DMPS headgroups respond to the changing field, causing the change in orientation of the ensemble of chains. The smaller response of the 1:1 mixture may be because the average area per molecule at the transfer pressure is slightly smaller–molecules would be more tightly packed than in the 9:1 mixture and this small difference in packing is enough to alter bilayer properties. The question then arises as to why the 1:1 mixture responds where pure DMPE does not and is most likely answered by considering the interaction between the DMPS headgroups and the negative surface charge. The interplay between chain–chain interactions and headgroup–headgroup and headgroup–field interactions is clearly complex. The tilt angles determined with in situ IR (at low charge densities) are larger than would be expected for monolayers in a solid phase. (Below we see that GIXD measurements indicate perpendicular chains for the solid phase.) This observation may suggest a small change in molecular orientation on transfer of molecules to the substrate, perhaps as a result of the interaction of the headgroup with the substrate. However, the difference is minor and the IR peak positions and widths indicate that the extent of ordering remains very high when monolayers are transferred, so we conclude that there is no significant change in structure on transfer of the monolayers.

#### 3.3.2 Ester Carbonyl Groups

**Figure 5:**
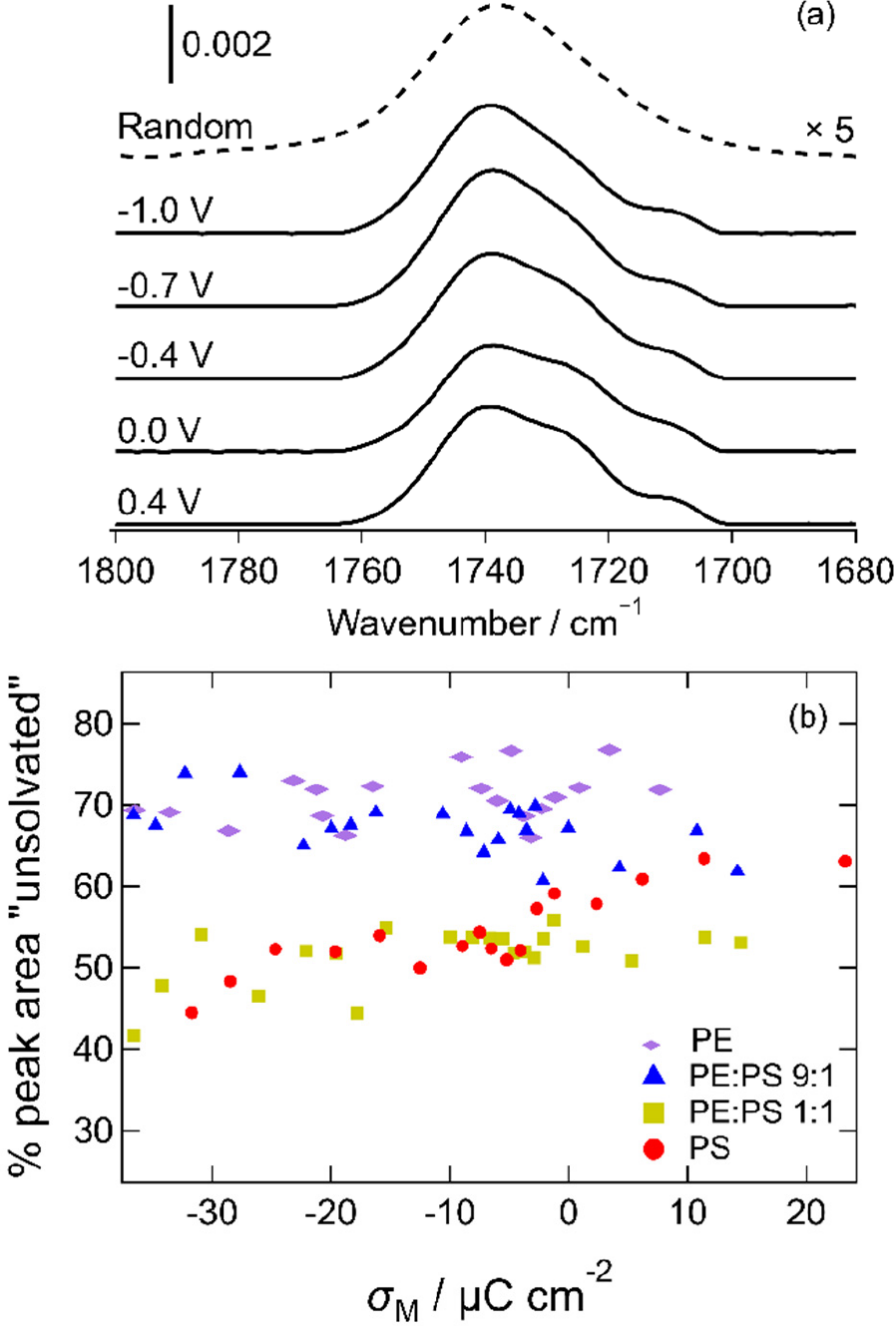
(a) Selected IR spectra of the 9:1 mixture in the C=O stretching region at the indicated potentials. The dashed line is the calculated spectrum for randomly oriented molecules. (b) Plot of the proportion of the peak area corresponding to the 1740 cm^–1^ component as a function of charge. Data for DMPE and DMPS are reproduced from refs (53) and (54), respectively, with permission. Copyright 2013 and 2014, the authors under a Creative Commons (CC-BY) license.

Figure 5a presents IR spectra in the C=O stretching region of the spectrum, acquired for the 9:1 mixture at a series of applied potentials. The band is normally composed of two or three contributions, depending on the degree of solvation of the lipids or the presence of various cations.^95,98,100^ In these spectra, the bands are each fitted to three peaks. The highest-wavenumber band (∼1740 cm^–1^) is associated with carbonyl groups that are not partaking in hydrogen bonding, and the lower-wavenumber peaks are associated with carbonyl groups that are involved in hydrogen bonding with water (for PS, the lowest-wavenumber vibration has also been suggested to result from hydrogen bonding with ammonium groups of neighboring molecules).^95,98,100^ The shapes of the peaks in the spectra suggest that the mixed bilayers contain more solvent than DMPE and less solvent than DMPS. By dividing the area of the 1740 cm^–1^ peak by the total area of the two or three peaks, an estimate of the degree of solvation of the ester region of the lipid bilayer can be obtained.^53,54^ (This value represents an average over the two halves of the bilayer.) This proportion is plotted for each bilayer as a function of charge density in Figure 5b. Data for DMPE^53^ and DMPS^54^ are included for comparison. This plot shows that the bilayers have similar hydrogen bonding interactions at small charge densities. At negative charge densities, the hydrogen bonding interactions of the 9:1 mixture are similar to those of DMPE, while those of the 1:1 mixture are similar to those of DMPS. Apparently, the tendency of the bilayer to incorporate water as the charge density increases is dependent on the DMPS content: the higher the DMPS content, the more solvent is incorporated into the headgroups. This is likely to be related to the greater need of the anionic headgroups to be screened from the negative charge density on the metal.^54^ Bilayers of the zwitterionic molecule DMPC, which have a stronger response to the electric field than DMPE bilayers, decrease in solvent content at negative charge densities.^51^ This behavior was attributed to the egress of solvent from the bilayer to form a cushion between the bilayer and the surface,^45,49^ based on complementary neutron reflectivity studies on similar bilayers.^45,49^ The combination of the electrochemical and IR data in the present work suggests that the DMPE:DMPS 9:1 mixture is a more flexible version of a DMPE bilayer, more able to respond to an electric field, while the 1:1 mixture is intermediate between DMPE and DMPS, with a smaller response and with similar solvent content to DMPS bilayers.

The differences in behavior between the mixtures and their individual components were investigated further using X-ray and neutron reflectivity and X-ray diffraction (vide infra). Specifically, the aims of these experiments were (i) to ascertain whether packing of the mixtures was looser than that of DMPE itself, which might explain their ability to respond to the application of an applied field, and (ii) to determine whether the monolayers used for deposition had different headgroup solvation, which might be incorporated into the deposited bilayer. Alternatively, a trend in solvation might reveal different tendencies for the bilayers to incorporate solvent. The treatment of the IR spectra above gives an indication of solvation that is useful for identifying trends and comparing bilayers, whereas reflectivity methods give direct information on the volume fraction of solvent contained within the headgroup region.

### 3.4 Brewster Angle Microscopy

**Figure 6:**
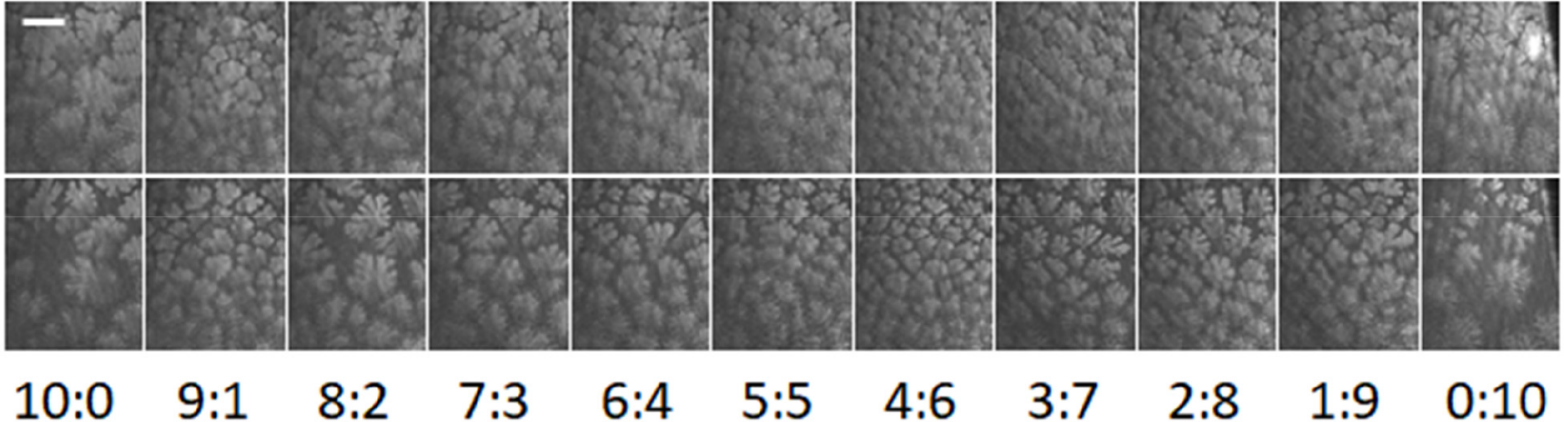
BAM images acquired at the indicated PE:PS molar ratios. Top 46 Å^2^, bottom 51 Å^2^. The white bar represents 20 μm, and the scale is the same in each image.

BAM was used to investigate further the differences in isotherms of the mixtures at lower surface pressures, where the liquid expanded and liquid condensed phases coexist. Selected BAM images are presented in Figure 6 for each composition at two different molecular areas (46 and 51 Å^2^). These images show extended condensed phase regions within the fluid phase for DMPE and DMPS, similar to structures reported previously for DMPE monolayers.^101^ Smaller structures have been reported for DMPS^102^ (but at higher temperature than in our measurements), which are similar to some of our structures for DMPS-rich monolayers at higher molecular area. The condensed regions are branched, an indication of relatively fast growth kinetics and low line tension between the two phases.^103^ For mixtures, the domain size is smaller and the extent of branching (or length of the branches) less. It was suggested for DMPE, based on the formation of fractal-like structures and the length of time required for them to relax to compact domains, that the line tension was weak or the DMPE molecules had low mobility.^101^ McConnell and co-workers have reported that the shapes of domains can be predicted by considering the balance between intermolecular repulsions (from aligned dipoles) and line tension,^104,105^ and Andelman et al. extended the theory to include a consideration of charged molecules (in the limits of low and high screening of charge by the subphase medium).^106^ The fingers of the DMPS domains are less rounded than those of the DMPE domains, which may be a result of greater repulsion between molecules, but the mixtures do not display a trend in shape from DMPE to DMPS. Instead, as the DMPS content is increased, the size of the domains and the length of the branches decrease to a minimum at 60% DMPS and then increase again. The relatively small size of the domains of the nearly equimolar mixtures suggests relatively higher rates of nucleation or slower rates of growth than for mixtures with a majority of one or the other lipid.

### 3.5 Grazing Incidence X-ray Diffraction

**Figure 7:**
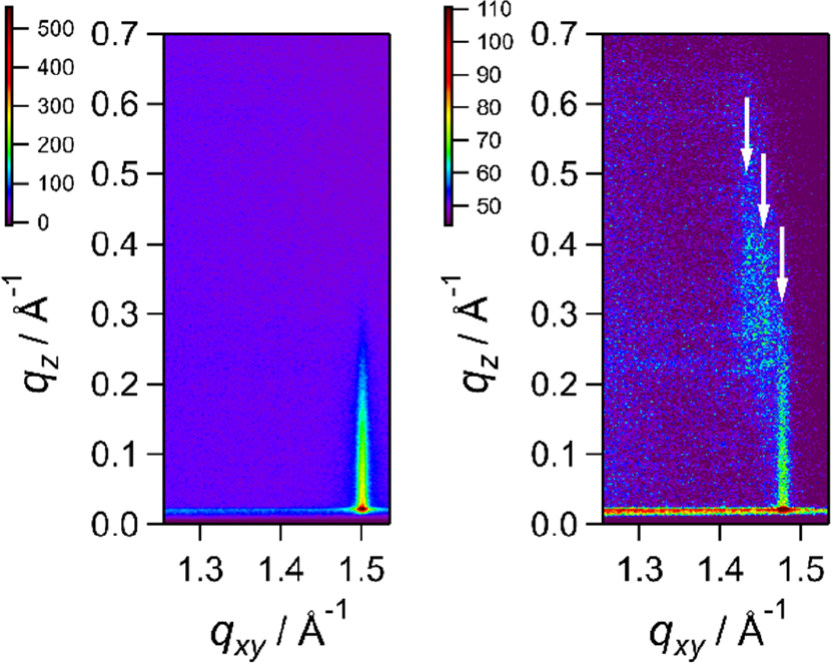
Representative GIXD images for the 1:1 mixture. Left: obtained at 47 mN m^–1^. Right: obtained at area A2.

**Figure 8:**
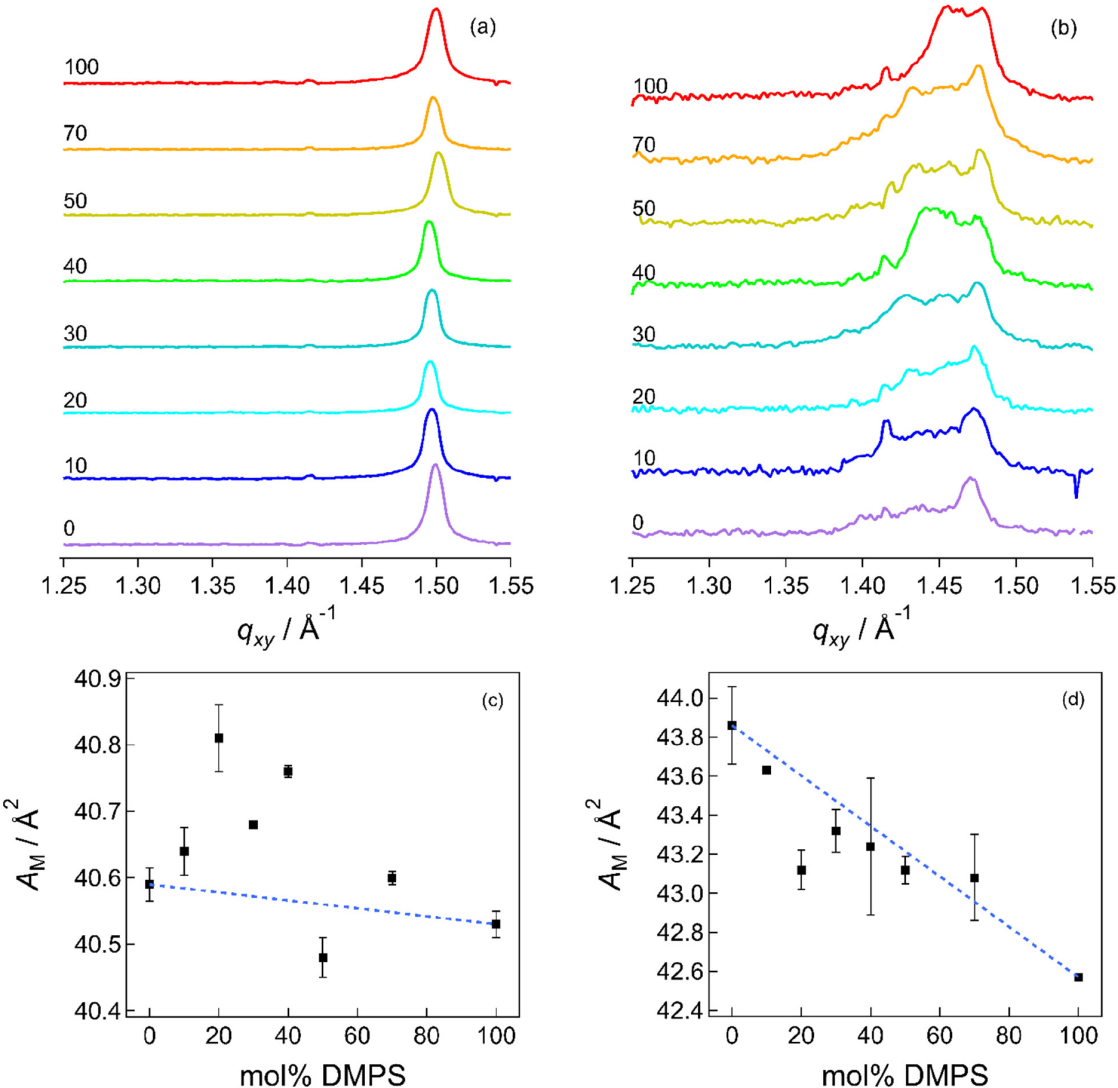
Bragg peaks for (a) 47 mN m^–1^ and (b) area A2 (nominally 42 Å^2^). The annotations indicate the mol % of DMPS. (c) and (d) Corresponding areas per molecule determined from GIXD peak positions. Dashed lines represent the molecular areas for ideal mixtures.

Figure 7 shows representative GIXD of the 1:1 mixture of DMPE and DMPS, acquired at two different points on the isotherm: 47 mN m^–1^ and 42 Å^2^ (area A2). The single peak in the image at 47 mN m^–1^ indicates that hydrocarbon chains are organized in a hexagonal arrangement, where the *d* spacing of the {1 1}, {1 0}, and {0 1} sets of planes is the same.^107^ At area A2, the chains are tilted, the hexagonal unit cell is distorted, and the degeneracy is broken. The positions of the peaks can be determined and analyzed to provide information on the interchain spacing and tilt angle from the surface normal.^107^ An explanation of the analysis used is given in the Supporting Information. Plots of integrated intensity vs *q*_*xy*_ for each composition are presented in Figure 8 for these points on the isotherm, along with corresponding plots of derived molecular area vs composition. (Plots for other pressures and molecular areas are provided in the Supporting Information, Figure S9.) For each composition, a single Bragg peak is observed at 47 and 40 mN m^–1^. At area A1, a single peak is observed for every monolayer except for pure DMPE, where two peaks are observed. At area A2, three peaks are observed, whose positions are consistent with an oblique unit cell formed of tilted hydrocarbon chains.^107^ At a larger area per molecule, diffraction was weak, if present at all, and typically only a weak feature corresponding to the {1 1} *d* spacing was observed, suggesting very little long-range order as chains tilt farther from the surface normal. Unit cell parameters derived from fitting the Bragg peaks (to a Voigt function) are given in Tables S1–S8 in the Supporting Information. In the solid phase, the areas are similar across the composition range, as was observed in isotherm data, and the values for DMPE are similar to those reported by Helm et al.^18^ However, the plot of area vs composition in Figure 8 does show a small positive excess area for all compositions except 1:1, which has a small negative excess area. The trend bears a resemblance to the excess energy results and may be explained by small disruptions to the hydrogen bonding network on mixing (vide supra). Although the differences are small, it is telling that similar results are observed using such different techniques: one a macroscopic thermodynamic measurement and the other a direct structural measurement. The slightly closer packing observed for the 1:1 mixture in each case may explain the weaker response to the applied electric field than for the 9:1 mixture that was observed in the IR spectra.

At area A1, DMPE is in the L_c_ phase, at a lower surface pressure than the other monolayers. The observation of tilting of hydrocarbon chains at this molecular area is consistent with a monolayer in the L_c_ phase. The larger calculated area per molecule (42.2 Å^2^ compared with 41.1 Å^2^ for PS) reflects the tilt of the chains; the cross-sectional area of the chains matches the molecular areas determined for the other compositions. The molecular areas of most of the different compositions are similar. At area A2, it can be observed that the areas per molecule and chain tilt angle for the mixtures decrease from DMPE to DMPS (Figure S10 in the Supporting Information), although, again, the changes are small. There is more variation in packing at this lower surface pressure than at the higher pressures. At this area per molecule, there is a variation in the slopes of the isotherms, as the surface pressures of the plateaux are lower for DMPS-rich samples than for DMPE-rich samples. There may be different degrees of condensation, and the isotherm area is an average of the monolayer while diffraction indicates the packing of ordered phases only. Only weak diffraction was observed at area A3. Three peaks could be observed for three compositions, but only the {1 1} reflection was observed for others, with low intensity. The molecular areas derived from the images with three peaks were around 44 Å^2^, similar to the isotherm molecular area. No diffraction was observed for the highest area, A4. GIXD was also measured for the deuterated lipids at the compositions used in the NR measurements to ensure that the structures were comparable. There is little difference in the data between deuterated and undeuterated lipids at 40 mN m^–1^. At area A1, the deuterated 9:1 mixture showed some distortion of the peak, which likely corresponds to a slight tilting of the chains (∼9°). A slight tilt was also observed for deuterated DMPS, but the results for DMPE and for the 1:1 mixture were similar to those of undeuterated samples. Deuterated lipids generally gave peaks of slightly lower intensity, suggesting a lower degree of ordering.

### 3.6 X-ray and Neutron Reflectivity

**Figure 9:**
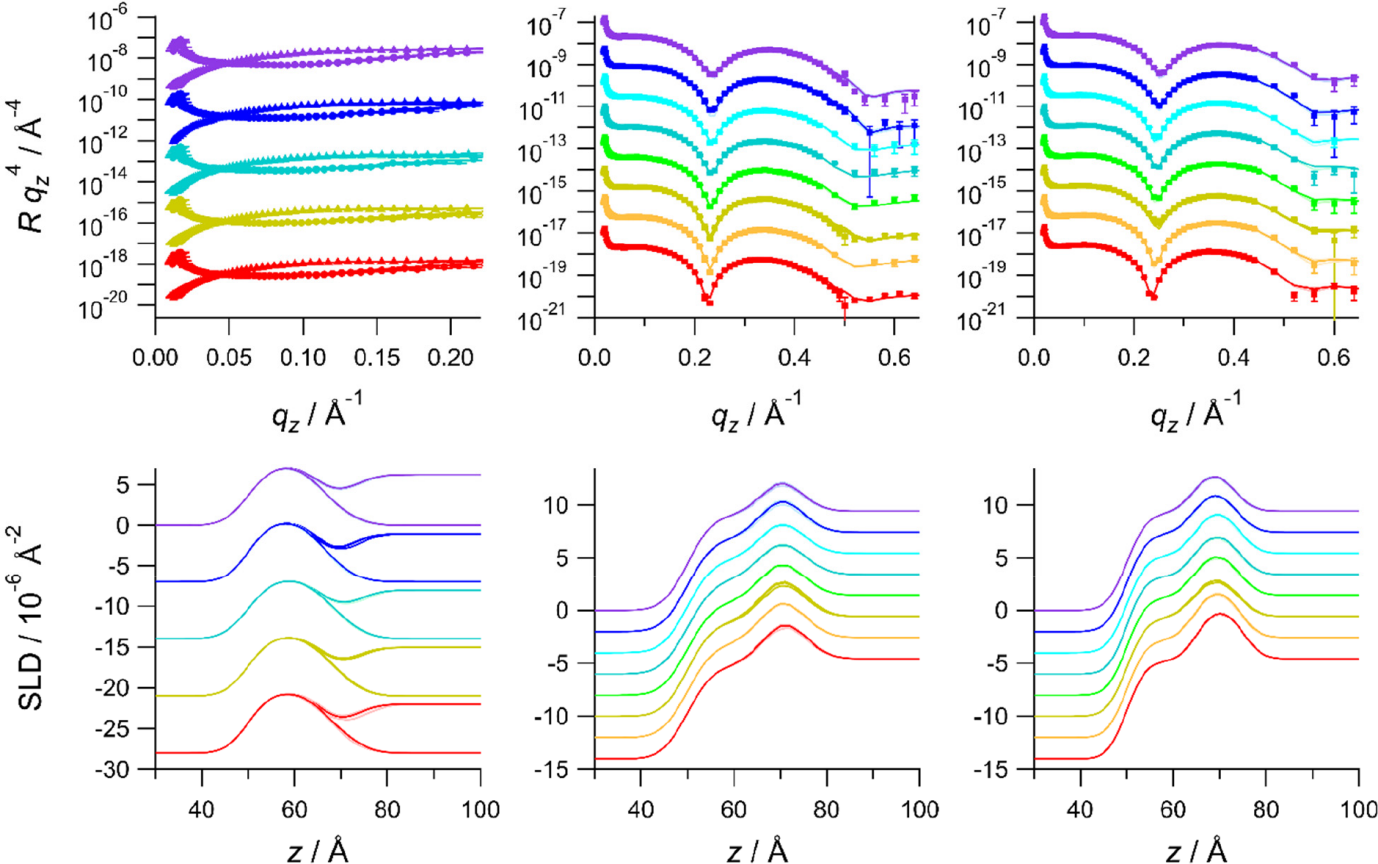
Reflectivity curves and fits (top) with corresponding SLD profiles (bottom) for NR at area A1 (left), XRR at 47 mN m^–1^ (middle), and XRR at area A2 (right). NR data are presented for (top to bottom) PE, PE:PS 9:1, 7:3, and 5:5, and PS. The ACMW subphase data are plotted as triangles, and the D_2_O subphase data are plotted as circles. XRR data are presented for (top to bottom in each case) PE, PE:PS 9:1, 8:2, 7:3, 5:5, and 3:7, and PS. The data have been offset for clarity using equal increments. Lines represent fits to the data, and shading represents the 95% confidence range.

NR was measured for a subset of the points on the isotherm: at 40 mN m^–1^, area A1 and area A4 for DMPE, DMPS, and the 9:1, 7:3, and 1:1 mixtures. NR data acquired at area A1 are given in the left panels of Figure 9. The top panel shows the reflectivity data, and the bottom panel shows the SLD profiles resulting from the fits. The data shown correspond to two contrasts: deuterated lipids on D_2_O and deuterated lipids on ACMW. The middle and right panels of Figure 9 present the XRR data and SLD profiles for the measurements at 47 mN m^–1^ and area A2, respectively. The remaining data are given in the Supporting Information. Data were fitted using a two-slab model comprising a headgroup slab and a tailgroup slab. The headgroup slab included the carbonyl groups of the ester linkages; the tailgroup slab contained the remaining carbon and hydrogen (or deuterium) atoms in the chains. It is generally assumed that deuterated and undeuterated samples do not differ in structure, at least in the solid and L_c_ phases, and it is common practice to cofit NR data for multiple contrasts including deuterated and undeuterated lipids; on occasion, this practice is explicitly considered and justified.^108^ To determine whether our NR data for deuterated samples and XRR data for undeuterated samples could be compared, XRR data were also acquired for some of the deuterated systems used in NR experiments, since X-rays are not sensitive to isotopic substitution. The results were not different at 40 mN m^–1^ in the solid phase. At area A1, small differences were observed between deuterated and undeuterated samples for the 1:1 and DMPS samples, but the DMPE-rich samples were very similar. Problems with beam damage were experienced for some of the lower pressures, and time constraints precluded our investigating differences in these structures more thoroughly. However, the different positions of the reflectivity minima do indicate differences between deuterated and undeuterated samples. This will be followed up in a future study.

For each composition, the minimum in the reflectivity observed in the XRR data becomes shallower and shifts to higher *q*_*z*_ as the monolayer is expanded to low surface pressure. This behavior indicates that the monolayer becomes thinner and headgroups less dense at these higher molecular areas, consistent with the observation of increasing tilt angle of the hydrocarbon chains. At a given pressure, there is a small shift in the position of the minimum as the DMPS content is increased, which indicates a small increase in monolayer thickness with increasing DMPS content.

The parameters used to describe the model were the scattering length densities (SLDs) of each slab, the thicknesses of each slab, and the roughness. The roughness was kept the same for each interslab interface.^108^ The data were fitted with the roughness fixed (for different values of the roughness) and then with the tailgroup slab SLD also fixed, for a range of different values of SLD that corresponded to 5 or 10 Å^3^ increments in molecular volume. The data were fitted in RasCal^79^ using the Bayesian MCMC algorithm. Areas per molecule from the diffraction measurements (where diffraction was observed) or from the isotherms (if diffraction was not observed) were used to assess the acceptability of the fit. The details of the procedure adopted are explained in the Supporting Information. In general, fitting of the NR data yielded higher molecular areas for the tailgroup slab than the XRR data but the tailgroup slab thicknesses were similar. (It should be borne in mind that there is potential for variation in the scattering length if deuteration is imperfect and using a lower scattering length would reduce the calculated molecular area values.) It was possible to obtain good fits to the XRR data with molecular areas in good agreement with GIXD or isotherm results.

For the headgroup slab, the solvated headgroup volume was obtained from the tailgroup molecular area and the headgroup slab thickness, as the molecular area must be the same in each slab. This headgroup volume contains a contribution from the volume of the “dry” or unsolvated lipid headgroup and the water molecules solvating it. Ideally, the SLD could then be used to calculate the volume fraction of water from the SLDs of water and the unsolvated headgroups. Unfortunately, independent values for headgroup volume are not always available in the literature or there are conflicting values. Moreover, the average headgroup volume in a mixture may not always be the same as the theoretical weighted average of the two components, although our diffraction data suggest that this may not be a problem for DMPE:DMPS mixtures. In the case of NR data, different subphase contrasts can be used to help to distinguish a change in headgroup volume from a change in solvent content (and to calculate the two parameters). In the present case, this process is complicated by the ability of the ammonium protons to exchange with D_2_O in that subphase^62^ and so alter the average scattering length of the dry headgroup, although most studies appear to assume a scattering length based on no H/D exchange. With three unknown parameters and two subphase contrasts, it is not possible to determine unambiguously the solvent volume fraction. Therefore, we have assumed a solvated headgroup volume from the tailgroup area and headgroup slab thickness and used that in conjunction with the SLD from the D_2_O measurement and the scattering lengths of lipid and solvent to obtain a volume for the unsolvated headgroup and the number of water molecules per lipid. Similar calculations were carried out using the molecular area determined independently from GIXD or isotherm measurements. The SLD from the ACMW measurement was then used to confirm that the molecular area for this fit was in reasonable agreement with that used in the D_2_O calculation. For XRR data, a similar calculation to that used for the D_2_O parameters was carried out. These calculations are explained in the Supporting Information. Solvation levels for the D_2_O and XRR calculations were found to be comparable where up to one headgroup hydrogen atom was assumed to exchange with deuterium. When a headgroup scattering length corresponding to greater numbers of exchanged hydrogen atoms was used, unphysical values of water content resulted, so we conclude the level of exchange to be low in these measurements.

The structural parameters obtained for XRR data of DMPE are similar to those reported by Helm et al. for DMPE monolayers in the L_c_ and solid phases,^18^ and the trends with surface pressure or molecular area are very similar. The tailgroup slab increases in thickness, as the tilt angle of chains decreases, and the headgroup slab decreases in thickness. That observation was attributed to a flattening of the headgroup on compression.^18^ At lower surface pressures, the solvent content of the headgroup slab increases, as the monolayers are more expanded with larger average molecular area. In the solid phase, the water is squeezed out of the headgroup slab. Roughness increases upon compression, as headgroups may stagger in order to maximize chain packing in the tailgroup slab. Similar general behavior has been reported for DMPS,^63^ but those measurements were carried out on an electrolyte subphase, which alters the structure of the monolayer at higher molecular area, and so the values are not necessarily directly comparable. All of the mixtures displayed similar behavior to the pure components on compression. NR data are relatively insensitive for the headgroup slab but suggested a greater slab thickness for DMPS layers than for other samples. In the XRR data, a weak tendency was observed for the headgroup slab thickness to increase with increasing mole fraction of DMPS (Figure S29). As the molecular areas are similar, this increase in thickness results from an increase in headgroup molecular volume. At higher molecular areas, the increase in molecular volume with DMPS content is a result of an increase in solvation. The unsolvated headgroup volume appears to change little at these areas. For DMPE, the value obtained is ∼250–260 Å^3^ at these areas, close to that reported by Marsh,^109^ and for DMPS, a value of ∼250 Å^3^ is obtained, similar to that reported by Petrache^110^ and used by Campbell et al. to fit NR data obtained for DMPS at 10 mN m^–1^.^108^ Hence, the DMPE and DMPS headgroups are of comparable size and the average size of the headgroups varies little with composition. The anionic DMPS headgroups attract more solvent, to screen the charges, and the solvated volume increases, particularly in the range of up to 30 mol % DMPS. In the solid phase, the situation is different; the monolayers contain relatively little solvent as there is little capacity to accommodate it. Yet the average solvated headgroup volume still increases and appears to be a result of an increase in DMPS headgroup volume to values approaching those reported by Pan et al.^65^ (278 Å^3^) or Fragneto et al.^64^ (321 Å^3^). The effect is observed in DMPS-rich samples and appears to be counterintuitive, but it was not possible to obtain acceptable fits for solid phase samples using an unsolvated headgroup volume of ∼244 Å^3^ or for the more expanded phases with the larger volume of ∼305 Å^3^. We tentatively suggest a different DMPS headgroup conformation in the solid phase from the L_c_ phase to accommodate interlipid hydrogen bonding interactions between headgroups (cf. the lower density of ice than water).

The similarity in headgroup slab solvation at higher pressures appears at first sight to be inconsistent with the differences in the IR spectra of the supported bilayers. The appearance of the IR spectra suggested a lower solvation of DMPE than DMPS^54^ and intermediate solvation of the mixtures. Some reports attribute the lowest-wavenumber band (1710 cm^–1^) to hydrogen bonding interactions with other headgroups rather than to water; a similarity in solvent content between DMPE, DMPS, and mixtures would support that assignment. Alternatively, the structure of a monolayer may alter slightly on transfer to the substrate, perhaps if there is a specific interaction between the headgroup and the substrate. However, the analysis in Figure 4 shows that the differences in the estimated proportion of non-hydrogen-bonding carbonyl groups are small at lower surface charge densities (the more positive potentials) and greater at negative charge densities. It is possible that the monolayers are transferred with similar solvent content, which is unaltered on the substrate in the absence of electrical perturbation. When the applied field is increased, there is a greater tendency for solvation in the DMPS-rich samples. The greater solvation of the DMPS-rich samples observed at low surface pressure (Figure S29) suggests a greater propensity for water to interact with the anionic headgroups, which could explain the greater degree of interaction with solvent when the supported bilayer is exposed to a strong electric field and/or the anionic headgroups experience greater repulsion from the negatively charged surface.

## 4 Conclusions

The effect of composition on lipid phase behavior in models of the inner leaflet of mammalian cell membranes has been studied. The inner leaflet contains predominantly PE and PS lipids; while these lipid types have been studied using various methods,^18,61−63,66,68,86,110^ few investigations of their mixtures or the effect of composition have been reported.^56,65^ In this study, structural information acquired for lipid monolayers has been used to explain the electrochemical phase behavior of bilayers of the corresponding compositions. Surface pressure–area isotherms of DMPE/DMPS mixtures show small positive excess areas at most compositions and a tendency for phase transitions to occur at lower surface pressure as the mole fraction of the anionic lipid DMPS is increased. Electrochemical measurements of supported bilayers indicate similar general electrochemical phase behavior at each composition but with a change in the potential of zero charge at ∼40–50 mol % anionic lipid (DMPS). In situ electrochemical infrared data show marked differences in the response of the different lipid bilayers to increasing electric field strength: as the surface becomes negatively charged, mixed bilayers show an increase in the hydrocarbon chain tilt angle, while DMPE bilayers appear unperturbed. On increasing the field strength further, the chains of mixed bilayers return to their original tilt angle, unlike DMPS bilayer chains, which remain tilted. The solvent content around the ester groups in DMPS and DMPE:DMPS 1:1 mixtures increases as the charge on the electrode is made more negative. X-ray and neutron reflectivity measurements of the corresponding monolayers show little variation in solvent content at the surface pressure used to deposit the bilayers (in the solid phase), but in the L_c_ phase, the monolayers with >30% anionic lipid contained more solvent than the monolayers with lower DMPS content. GIXD measurements gave small positive excess areas for most compositions, apart from the equimolar mixture, and slightly greater tilt angles for DMPE-rich monolayers for a given area in the L_c_ phase. Both GIXD and IR indicate highly organized lipid films, with only a minor increase in tilt angle on transfer to the substrate. Taken together, the data show that seemingly minor structural differences between monolayers can still result in strong differences in the behavior of the bilayers they are used to form. The differences in packing inferred from isotherm and diffraction data were on the limit of the experimental error, yet a mixture comprising 10% DMPS exhibited a stronger response to an externally applied electric field than both the pure DMPE and the 50% DMPS mixture. Similarly, there was no trend in the solvation of monolayers in the solid phase, but the bilayers showed different tendencies to take up water, which matched the data obtained for monolayers at lower surface pressure. This result shows that measurements over a range of surface pressures can be very helpful for understanding the behavior of supported bilayers. The surface pressure of the supported lipid bilayers (the area between the chronocoulometry curves in the presence and absence of lipid) is lower as the surface charge is made more negative, so the monolayers at lower surface pressure give useful insight into the abilities of the bilayers to take up water.

We note that several parameters appear to change at composition ∼40 mol % DMPS. The surface pressure of the L_c_–S phase transition in the isotherm decreases in a linear fashion to 40 mol %, at which point a decrease in the slope occurs. A similar trend is seen for the onset of the L_e_–L_c_ phase transition. The potential of zero charge for the bilayer-coated electrode changes at >30–40 mol % PS. The solvation of the L_c_ phase appears to change at ∼30–40 mol % DMPS, and the propensity for perturbed bilayers to take up solvent changes either side of this composition. PS lipids make up ∼15–33% of the cytosolic leaflet of a mammalian cell membrane, depending on the type of cell.^111^ The changes in properties we observe between ∼10 and ∼40 mol % DMPS may explain why cells restrict their PS content to that range. PS is required for the function of some proteins, and the bilayer containing ∼10 mol % DMPS is close in structure and barrier properties to pure DMPE but more flexible, while the bilayers containing >30–40 mol % have a greater propensity to incorporate water when perturbed by an external field. The uptake of solvent in the presence of an electric field, which could result from an ion gradient or even the charge asymmetry conferred by the asymmetric distribution of PS lipids themselves in the natural membrane, would damage the barrier integrity. Although the models used are simplified, they do show that bilayers within the range of 10–30 mol % PS appear to provide the best balance of properties for the cell membrane. The results highlight the value of investigating the phase behavior of an extended range of lipid compositions if we are to acquire a detailed understanding of structure–property relationships. They also illustrate the benefits of using complementary methods to understand membrane behavior: both Langmuir monolayer studies and electrochemical methods provide a means to control the charge density and/or surface pressure of a monolayer or bilayer. The structural information that can be acquired for monolayers over a range of pressure sheds light on the reasons for differences between bilayers exposed to strong electric fields. In turn, electrochemical and associated in situ structural methods can reveal profound differences in behavior when bilayers that initially appear to be similar in structure are perturbed by such fields.

## Supporting Information Available

Additional surface pressure–area isotherms, additional chronocoulometry data, additional IR spectra and analysis, additional GIXD data and analysis, and additional neutron and X-ray reflectivity data and analysis (PDF)
