# Supplementary material for: Effect of Anionic Lipids on Mammalian Plasma Cell Membrane Properties
Source: Langmuir. 2023 Feb 9;39(7):2676–91. doi: 10.1021/acs.langmuir.2c03161 (PMC9948536; doi:10.1021/acs.langmuir.2c03161)
Supplement: Supplementary file 1 — la2c03161_si_001.pdf [file la2c03161_si_001.pdf]

Supporting Information for:

Effect of Anionic Lipids on Mammalian Plasma Cell Membrane Properties

Alexandra L. Martin,[1] Philip N. Jemmett,[1] Thomas Howitt,[1] Mary H. Wood,[1†], Andrew W. Burley,[1] Liam R. Cox,[1] Timothy R. Dafforn,[2] Rebecca J. L. Welbourn,[3], Mario Campana,[3] Maximilian W. A. Skoda,[3] Joseph J. Thompson,[4] Hadeel Hussain,[4] Jonathan L. Rawle,[4] Francesco Carlà,[4] Christopher L. Nicklin,[4] Thomas Arnold[3,4,5,6] and Sarah L. Horswell[1]**

1. School of Chemistry, University of Birmingham, Edgbaston, Birmingham, B15 2TT, UK

2. School of Biosciences, University of Birmingham, Edgbaston, Birmingham, B15 2TT, UK

3. ISIS Pulsed Neutron and Muon Source, Science and Technology Facilities Council, Rutherford Appleton Laboratory, Harwell, Oxfordshire, OX11 0QX, UK

4. Diamond Light Source, Harwell Science and Innovation Campus, Chilton, Didcot, Oxfordshire, OX11 0DE, UK

5. European Spallation Source ERIC PO Box 176, SE-221 00 Lund, Sweden

6. Department of Chemistry, University of Bath, Claverton Down, Bath, BA2 7AY, UK

** Corresponding authors: tom.arnold@ess.eu, s.l.horswell@bham.ac.uk*

[†] Present address: Department of Chemistry, University of Cambridge, Lensfield Road, Cambridge, CB2 1EW, UK

1. Surface Pressure-Area Isotherms

Additional isotherm data are presented in Figure S1. The surface pressures at the onset of the L_e–L_c phase transition and at the L_c–S phase transition are plotted in Figure S2 (left). Excess Gibbs energies of mixing at 47 mN m^{-1} are also plotted in Figure S2 (right).

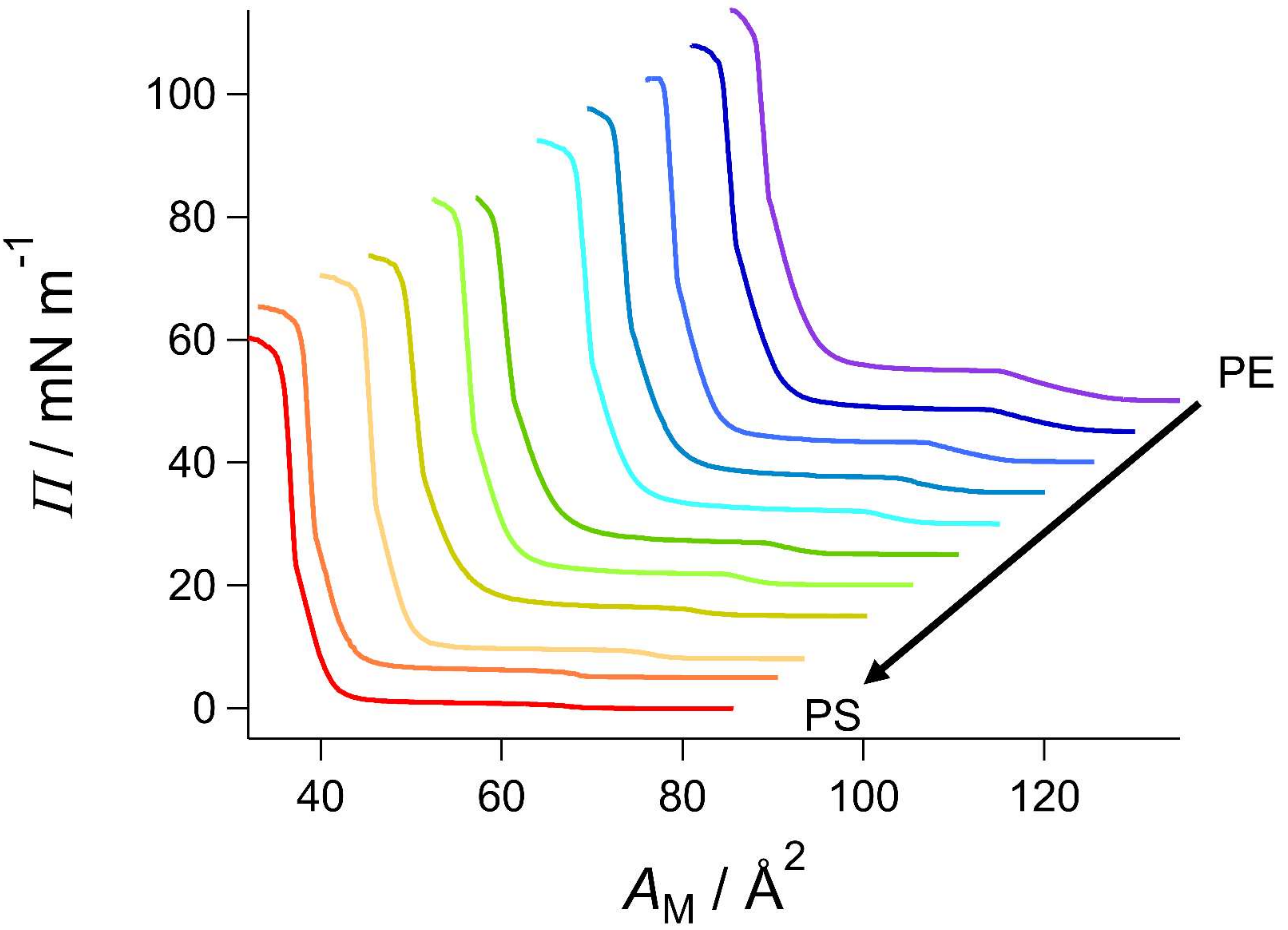

Figure S1. Isotherms of DMPE/DMPS mixtures recorded at 19 °C. Back (purple) to front (red): DMPE to DMPS with DMPS increasing in increments x_{PS}=0.1.

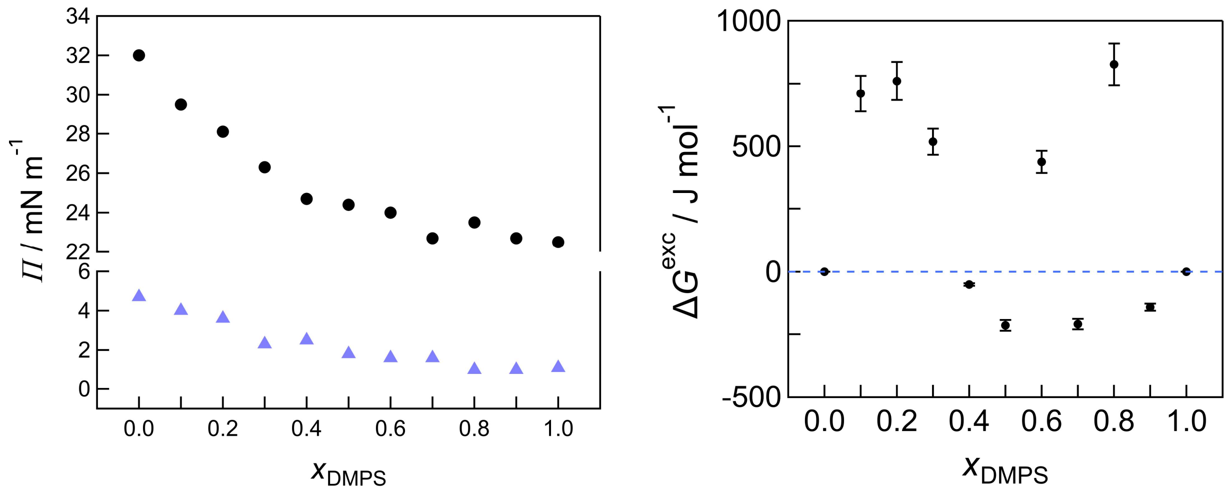

Figure S2. Left: Surface pressure at phase transition. Black squares L_c–S transition, blue triangles onset of L_e–L_c transition. Right: Excess Gibbs energies of mixing as a function of composition.

2. Electrochemical Data

Figure S3 presents additional chronocoulometry data for the remaining mixtures.

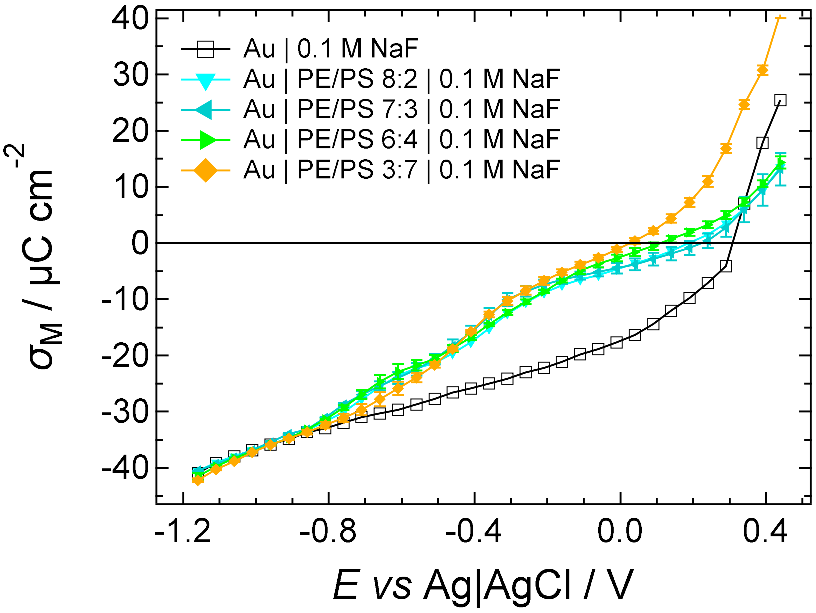

Figure S3. Chronocoulometry data for PE/PS mixtures at the indicated compositions (9:1 and 1:1 mixtures presented in main text, Figure 3a). Error bars represent standard deviation from three measurements.

3. PM-IRRAS Data Analysis

The integrated areas of the peaks were used to determine the orientation of the associated transition dipole moments. Following Lipkowski and co-workers, the absorbance is related to the angle between the transition dipole moment of the vibrational mode and the surface normal by the relation:[S1,S2]

$$\int A d\tilde{\nu} \propto \ |\boldsymbol{\mu} \cdot \boldsymbol{E}|^2 = |\mu|^2 \langle E \rangle^2 \ \cos^2 \theta \qquad \text{Eq. S1}$$

where A is the absorbance, $\tilde{\nu}$ the wavenumber, $\boldsymbol{\mu}$ the dipole moment, $\boldsymbol{E}$ the electric field (normal to the surface) and θ the angle made between the dipole moment and the surface normal. The intensity is also related to the quantity of material so must be compared with that obtained from a simulated spectrum of randomly oriented molecules, using the isotropic optical constants of the materials involved and the same experimental parameters as for the measurements (angle of incidence, thickness of bilayer and thickness of electrolyte between sample and window). It is then possible to calculate the angle of the transition dipole from the surface normal using Eq. S2:[S1-S3]

$$\cos^2 \theta = \frac{1}{3} \frac{\int_{\text{meas}} A d\tilde{\nu}}{\int_{\text{random}} A d\tilde{\nu}} \qquad \text{Eq. S2}$$

The angles of the CH_2 symmetric and asymmetric stretching modes are orthogonal to one another and to the direction made by the chain backbone. Once the angles of the dipole moments of these vibrational modes are known, the chain tilt angle can be calculated from Eq. S3:[S4]

$$\cos^2 \theta_{\text{s}} + \cos^2 \theta_{\text{as}} + \cos^2 \theta_{\text{ch}} = 1 \qquad \text{Eq. S3}$$

where θ_s and θ_{as} are the angles between the symmetric and asymmetric stretching modes and the surface normal, respectively, and θ_{ch} is the tilt angle between the hydrocarbon chain axis and the surface normal.

Selected spectra in the C–H stretching region for the DMPE:DMPS 1:1 mixture are presented in Figure S4. Figure S5 shows an example of fitting the peaks in the spectrum acquired for the 9:1 mixture at 0 V. Figure S6 presents the tilt angles of the C–H ν_{as} and ν_s stretching mode transition dipole moments and the chain tilt angle vs applied potential, for the 9:1 and 1:1 mixtures.

Figure S7 gives the spectra in the carbonyl C=O stretching region for the DMPE:DMPS 1:1 mixture. The asymmetric stretching mode of the DMPS carboxylate group is also expected in the spectral region ~1550–1680 cm^{-1} [S5,S6] but the weakness and breadth of the peaks prevented reliable subtraction of a background, so this mode could not be analyzed further.

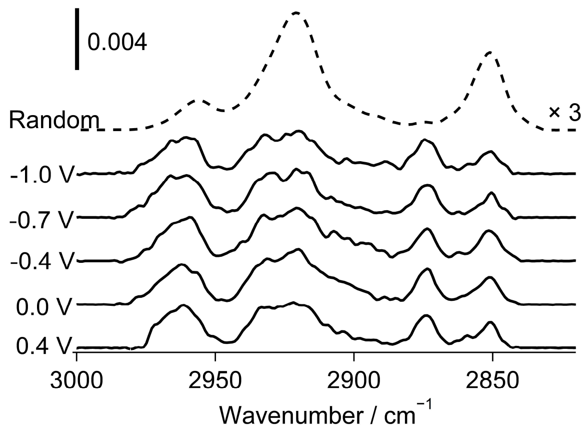

Figure S4. Selected spectra in C–H stretching region for the 1:1 DMPE:DMPS mixture, acquired at the indicated potentials. The dashed line is a simulated spectrum for the same experimental conditions as the measured spectra, using the isotropic optical constants of the constituent molecules.

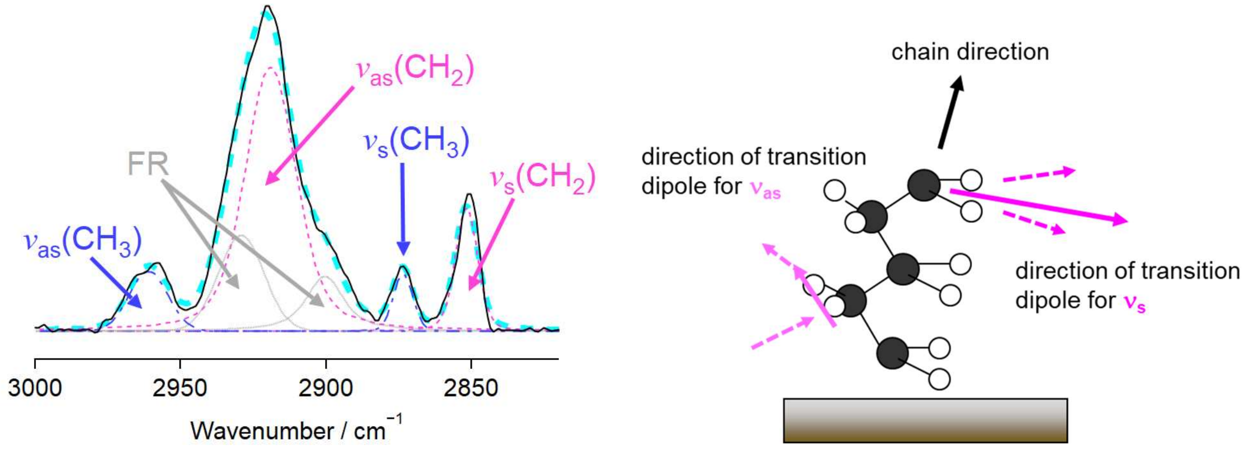

Figure S5. Left: example of fitting the spectra: spectrum acquired for PE:PS 9:1 at –0.4 V. Right: cartoon illustrating the relative directions of the vibrational mode transition dipole moments and the chain backbone.

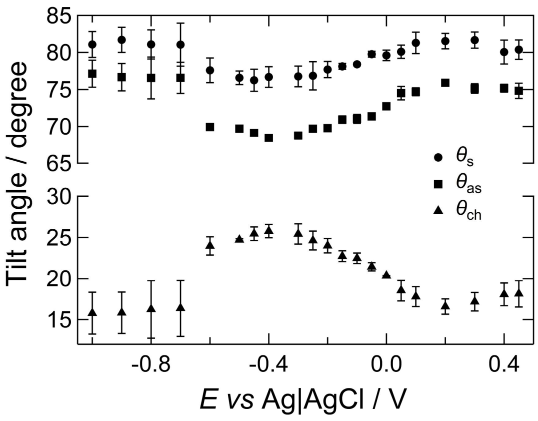

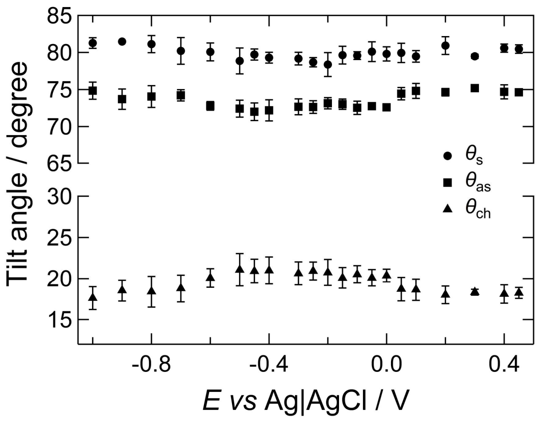

Figure S6. Plots of angle between C–H stretching mode transition dipole moments and the surface normal and of the resulting angle between the hydrocarbon chain and the surface normal, as a function of applied potential. Left: DMPE:DMPS 9:1. Right: DMPE:DMPS 1:1.

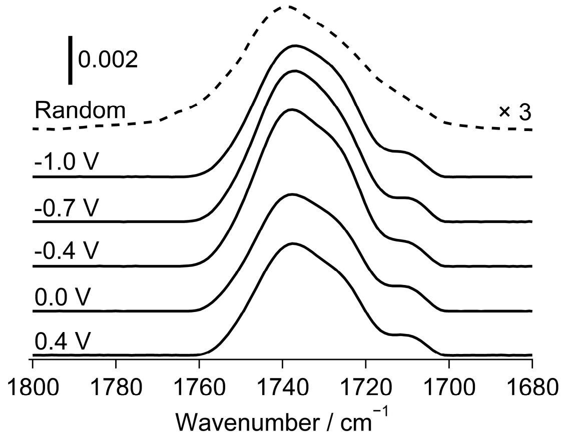

Figure S7. Selected spectra in C=O stretching region for the 1:1 DMPE:DMPS mixture, acquired at the indicated potentials. The dashed line is a simulated spectrum for the same experimental conditions as the measured spectra, using the isotropic optical constants of the constituent molecules.

4. Grazing Incidence X-ray Diffraction Data

Grazing incidence X-ray diffraction (GIXD) data were acquired as images on an array detector and scaled to q_{xy} and q_z using an in-house written MATLAB script. To facilitate analysis of the data, the script also interpolated the scaled images for an even step-size in q_{xy} and q_z.

Images show one peak where the chains are upright (or close to upright), indicating a hexagonal unit cell. Additional peaks at $q_z \neq 0$ are observed where chains are tilted.[S7] The positions of the peaks in *xy* and *z* were found to be consistent with a rectangular unit cell (in the case of two peaks) or an oblique unit cell (in the case of three peaks) and were analyzed accordingly. [S7] A sketch of the unit cell to indicate the structural parameters obtained is given in Figure S8. The lattice parameters were derived from fitting plots of the Bragg peaks, which were obtained by summing the intensities over a fixed q_z range for each value of q_{xy}. Similarly, plots for Bragg rods can be obtained by summing intensity over a fixed range of q_{xy}, for each value of q_z. The background intensity was subtracted and the peaks were fitted using a Voigt function in OriginPro. Additional plots comprising "slices" of the image were sometimes used to aid fitting. The spacing between lattice planes was determined from Eq. S4:

$$d_{xy,hk} = 2\pi/q_{xy,hk} \qquad \text{Eq S4}$$

and used to calculate in-plane lattice parameters and an area per chain, thence an area per molecule.

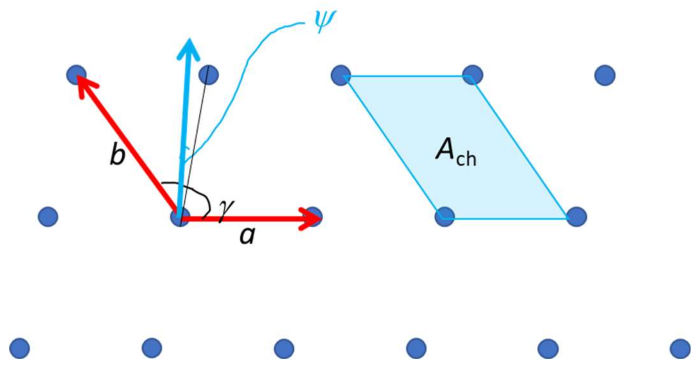

Figure S8. Structural parameters of the unit cell. The shaded area is the area occupied per chain (area per molecule is taken as twice this area).

The lattice parameters and areas per molecule are given in Tables S1–S8. The errors reported in q_{xy} are the standard deviations of peak position from between 2 and 10 images; the errors in lattice parameter and area are propagated from these. To estimate the tilt angle of the chains from the surface normal, θ, the relation[S8]

$$q_{z,hk} = (\boldsymbol{q_{xy,hk}} \cdot \hat{\boldsymbol{e}}) \tan(\theta) \quad \text{Eq. S5}$$

was used, where $\hat{\boldsymbol{e}}$ is the unit vector pointing along the chain tilt direction. (Note that this angle, θ, is commonly denoted t in GIXD literature; here we use θ for consistency with the notation used for PM-IRRAS measurements.) For oblique cells, the direction of the chain tilt (the angle ψ) is also determined from these calculations.

Representative Bragg peaks are plotted in Figure S9 for each surface pressure. For presentation purposes only, the peak for a measurement of DMPE or DMPS at 40 mN m^{-1} in each experiment has been used for scaling. Plots of area per molecule vs mol% DMPS at each pressure and nominal molecular area are provided in Figure S10.

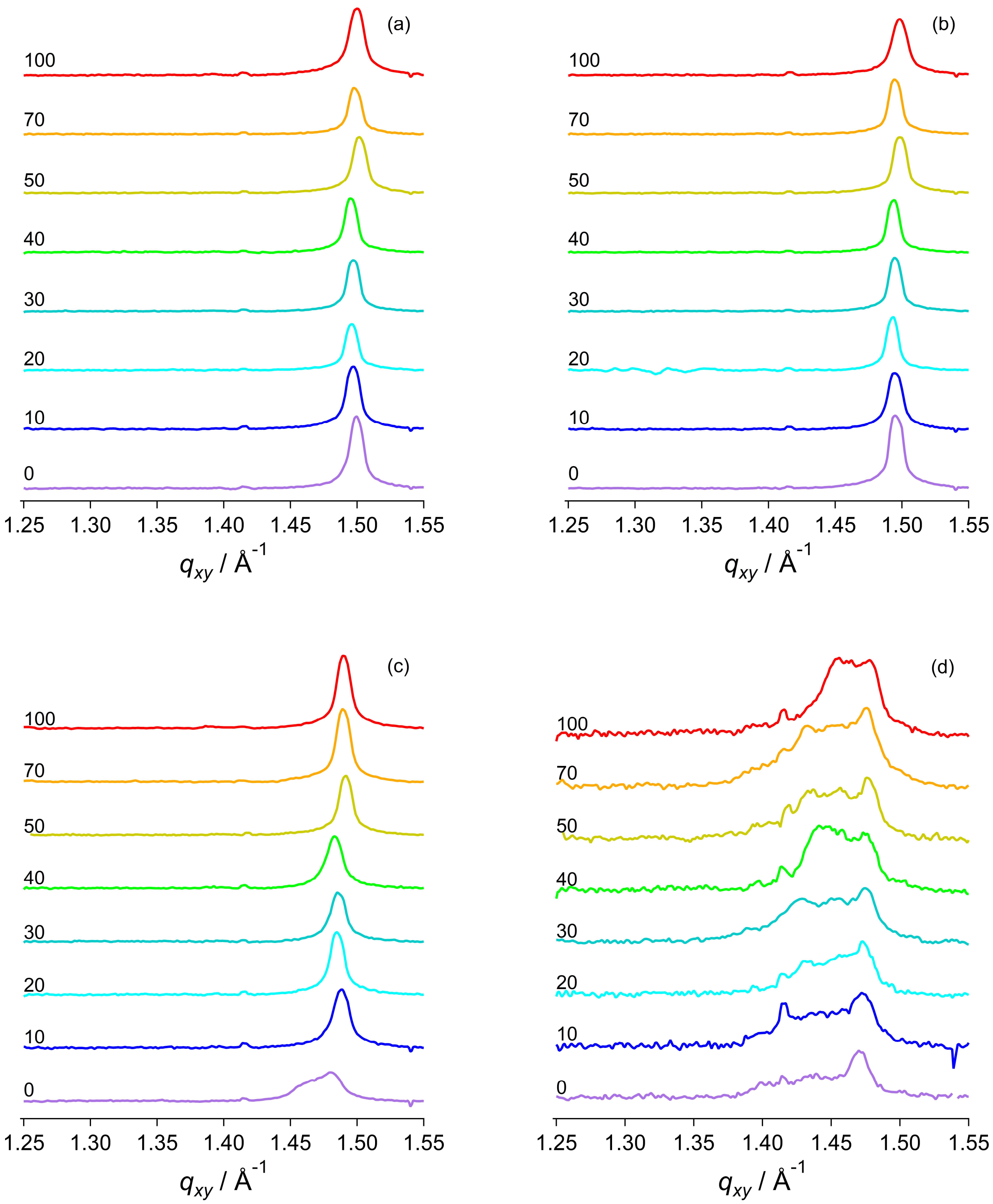
(a)
100
70
50
40
30
20
10
0
1.25 1.30 1.35 1.40 1.45 1.50 1.55
q_{xy} / Å^{-1}
(b)
100
70
50
40
30
20
10
0
1.25 1.30 1.35 1.40 1.45 1.50 1.55
q_{xy} / Å^{-1}
(c)
100
70
50
40
30
20
10
0
1.25 1.30 1.35 1.40 1.45 1.50 1.55
q_{xy} / Å^{-1}
(d)
100
70
50
40
30
20
10
0
1.25 1.30 1.35 1.40 1.45 1.50 1.55
q_{xy} / Å^{-1}

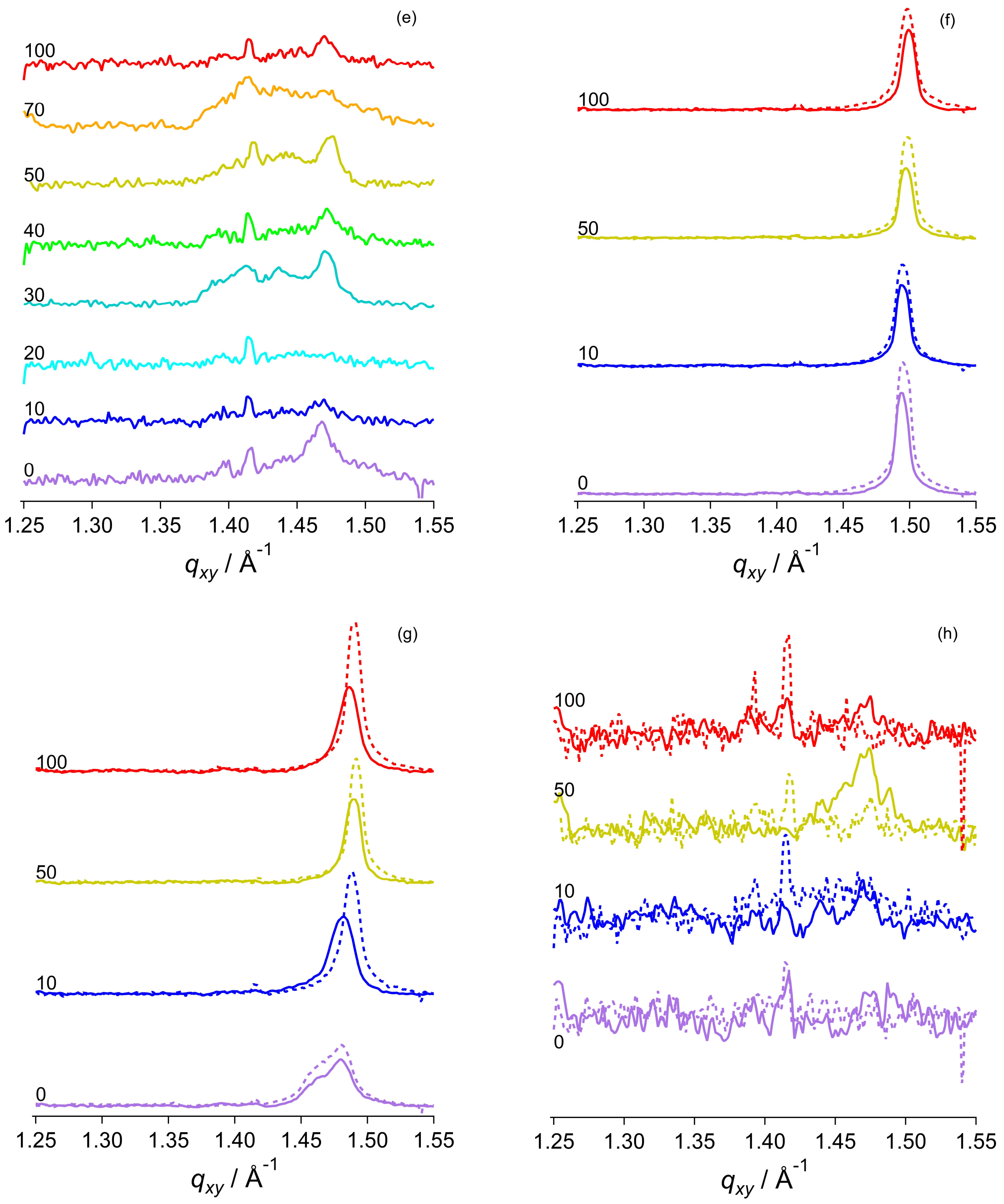

Figure S9. Bragg peaks for h-lipids at (a) 47 mN m^{-1}, (b) 40 mN m^{-1}, (c) 38 Å^{2}, (d) 42 Å^{2}, (e) 44 Å^{2} and for d-lipids compared with h-lipids at (f) 40 mN m^{-1}, (g) 38 Å^{2} and (h) 46 Å^{2}. Data for d-lipids are plotted as solid lines and for h-lipids as dotted lines.

Table S1. 47 mN m^{-1} h-lipids

% DMPS	q_{xy} / Å^{-1}	d / °	$a = b$ / °	γ / °	A_M / Å^2	θ / °	ψ / °
0	1.499 (0.0009)	4.193 (0.003)	4.841 (0.003)	120	40.59 (0.04)	0	0
10	1.498 (0.001)	4.195 (0.004)	4.844 (0.004)	120	40.64 (0.07)	0	0
20	1.495 (0.002)	4.204 (0.006)	4.854 (0.006)	120	40.82 (0.11)	0	0
30	1.497 (0.0002)	4.197 (0.0004)	4.846 (0.0005)	120	40.68 (0.01)	0	0
40	1.496 (0.0003)	4.201 (0.0009)	4.851 (0.001)	120	40.76 (0.02)	0	0
50	1.501 (0.001)	4.186 (0.003)	4.834 (0.003)	120	40.48 (0.05)	0	0
70	1.498 (0.0003)	4.193 (0.0008)	4.842 (0.001)	120	40.60 (0.02)	0	0
100	1.500 (0.0009)	4.189 (0.002)	4.837 (0.003)	120	40.53 (0.05)	0	0

Table S2. 40 mN m^{-1} h-lipids

% DMPS	q_{xy} / Å^{-1}	d / Å	$a = b$ / Å	γ / °	A_M / Å^2	θ / °	ψ / °
0	1.496 (0.0008)	4.201 (0.002)	4.850 (0.003)	120	40.75 (0.04)	0	0
10	1.495 (0.0007)	4.202 (0.002)	4.852 (0.002)	120	40.78 (0.04)	0	0
20	1.493 (0.0005)	4.209 (0.001)	4.861 (0.002)	120	40.92 (0.03)	0	0
30	1.494 (0.0004)	4.204 (0.001)	4.855 (0.001)	120	40.82 (0.02)	0	0
40	1.494 (0.0001)	4.206 (0.0003)	4.857 (0.0004)	120	40.86 (0.01)	0	0
50	1.498 (0.0003)	4.193 (0.0008)	4.842 (0.001)	120	40.61 (0.02)	0	0
70	1.495 (0.0006)	4.201 (0.002)	4.851 (0.002)	120	40.77 (0.04)	0	0
100	1.498 (0.001)	4.195 (0.003)	4.845 (0.003)	120	40.65 (0.05)	0	0

Table S3. 40 mN m^{-1} d-lipids

% DMPS	q_{xy} / Å^{-1}	d / Å	$a = b$ / Å	γ / °	A_M / Å^2	θ / °	ψ / °
0	1.494 (0.0006)	4.206 (0.002)	4.857 (0.002)	120	40.85 (0.03)	0	0
10	1.495 (0.0006)	4.202 (0.002)	4.852 (0.002)	120	40.78 (0.03)	0	0
50	1.498 (0.0002)	4.196 (0.0006)	4.845 (0.0007)	120	40.65 (0.01)	0	0
100	1.498 (0.003)	4.195 (0.008)	4.844 (0.009)	120	40.64 (0.15)	0	0

Table S4. 38 Å² h-lipids

% DMPS	q_{xy} / Å^{-1}	d / Å	$a = b$ / Å	γ / °	A_M / Å^2	θ / °	ψ / °
0	1.482 (0.002), 1.465 (0.002)	4.240 (0.004) 4.290 (0.007)	4.915 (0.007)	119.2 (0.01)	42.17 (0.09)	11.9 (0.5)	0
10	1.489 (0.002)	4.219 (0.006)	4.872 (0.007)	120	41.11 (0.11)	0	0
20	1.487 (0.002)	4.226 (0.006)	4.880 (0.007)	120	41.24 (0.12)	0	0
30	1.485 (0.002)	4.232 (0.006)	4.886 (0.007)	120	41.36 (0.12)	0	0
40	1.483 (0.0001)	4.236 (0.0002)	4.892 (0.0002)	120	41.45 (0.004)	0	0
50	1.490 (0.003)	4.218 (0.008)	4.870 (0.009)	120	41.08 (0.15)	0	0
70	1.488 (0.002)	4.223 (0.004)	4.876 (0.005)	120	41.19 (0.08)	0	0
100	1.489 (0.002)	4.220 (0.004)	4.873 (0.005)	120	41.13 (0.09)	0	0

Table S5. 38 Å² d-lipids

% DMPS	q_{xy} / Å^{-1}	d / Å	a / Å	b / Å	γ / °	A_M / Å^2	θ / °	ψ / °
0	1.480 (0.002) 1.467 (0.003) 1.453 (0.005)	4.245 (0.005) 4.284 (0.009) 4.325 (0.016)	4.902 (0.011)	4.949 (0.019)	119.1 (0.02)	42.40 (0.18)	12.1 (0.9)	9.0 (2.0)
10	1.486 (0.002) 1.479 (0.003)	4.228 (0.005) 4.249 (0.008)	4.890 (0.009)	4.890 (0.009)	119.7 (0.014)	41.55 (0.11)	9.3 (1.2)	0
50	1.491 (0.002)	4.215 (0.006)	4.868 (0.006)	4.868 (0.006)	120	41.04 (0.11)	0	0
100	1.488 (0.001) 1.480 (0.003)	4.223 (0.004) 4.244 (0.010)	4.885 (0.007)	4.885 (0.007)	119.7 (0.09)	41.46 (0.15)	10.1 (0.0)	0

Table S6. 42 Å² h-lipids

% DMPS	q_{xy} / Å^{-1}	d / Å	a / Å	b / Å	γ / °	A_M / Å^2	θ / °	ψ / °
0	1.472 (0.001), 1.441 (0.004), 1.414 (0.005)	4.268 (0.004) 4.360 (0.013) 4.444 (0.016)	4.935 (0.015)	5.030 (0.018)	117.9 (0.02)	43.86 (0.20)	18.5 (1.5)	13.4 (4.3)
10	1.473 1.447 1.418	4.265 4.341 4.432	4.922	5.025	118.1	43.63 (est. 0.17)	17.5	9.9
20	1.474 (0.0006) 1.456 (0.002) 1.432 (0.003)	4.261 (0.002) 4.314 (0.005) 4.387 (0.009)	4.915 (0.005)	4.998 (0.010)	118.6 (0.007)	43.12 (0.10)	15.5 (0.5)	10.4 (1.1)
30	1.475 (0.0008) 1.453 (0.0007) 1.426 (0.004)	4.260 (0.002) 4.325 (0.002) 4.405 (0.011)	4.916 (0.002)	5.007 (0.012)	118.4 (0.003)	43.32 (0.11)	16.2 (0.8)	11.3 (1.3)
40	1.474 (0.001) 1.452 (0.004) 1.431 (0.011)	4.262 (0.004) 4.327 (0.011) 4.391 (0.033)	4.924 (0.013)	4.997 (0.038)	118.5 (0.02)	43.24 (0.35)	15.3 (0.1)	9.1 (1.3)
50	1.477 (0.001) 1.454 (0.006) 1.433 (0.005)	4.255 (0.004) 4.322 (0.007) 4.384 (0.003)	4.917 (0.008)	4.988 (0.003)	118.5 (0.01)	43.12 (0.07)	15.0 (0.4)	10.2 (3.3)
70	1.475 (0.001) 1.455 (0.005) 1.435 (0.005)	4.259 (0.003) 4.318 (0.015) 4.380 (0.016)	4.918 (0.017)	4.989 (0.019)	118.6 (0.02)	43.08 (0.22)	15.6 (1.0)	9.7 (1.5)
100	1.478 1.464 1.449	4.251 4.292 4.336	4.908	4.959	119.0	42.57 (est. 0.17)	13.5	12

Table S7. 42 Å² d-lipids (only DMPS showing weak diffraction)

% DMPS	q_{xy} / Å^{-1}	d / Å	a / Å	b / Å	γ / °	A_M / Å^2	θ / °	ψ / °
100	1.475<br>1.455<br>4.317	4.317<br>4.317<br>4.420	4.905	5.021	118.3	43.36	not calc [a]	not calc[a]

Table S8. 44 Å² h-lipids

% DMPS	q_{xy} / Å^{-1}	d / Å	a / Å	b / Å	γ / °	A_M / Å^2	θ / °	ψ / °
0	1.469 (weak)	4.276						
10	1.469 (weak)	4.278				~44		
20	1.472 (weak)	4.268						
30	1.471<br>1.439<br>1.407	4.271<br>4.367<br>4.467	4.934	5.048	117.8	44.08 (est. 0.17)	18.4	15.2
40	1.472 (weak)	--	--	--	--	--	--	--
50	1.474 (0.0002)<br>1.443 (0.002)<br>1.412 (0.002)	4.263 (0.0007)<br>4.353 (0.005)<br>4.450 (0.006)	4.924 (0.006)	5.034 (0.007)	117.9 (0.01)	43.82 (0.08)	not calc[a]	not calc[a]
70	1.471<br>1.437<br>1.410	4.271<br>4.373<br>4.410	4.942	5.037	117.8	44.06 (0.18)	not calc[a]	not calc[a]
100	1.471 (weak)	4.272	--	--	--	--	--	--

[a] Uncertainty too high.

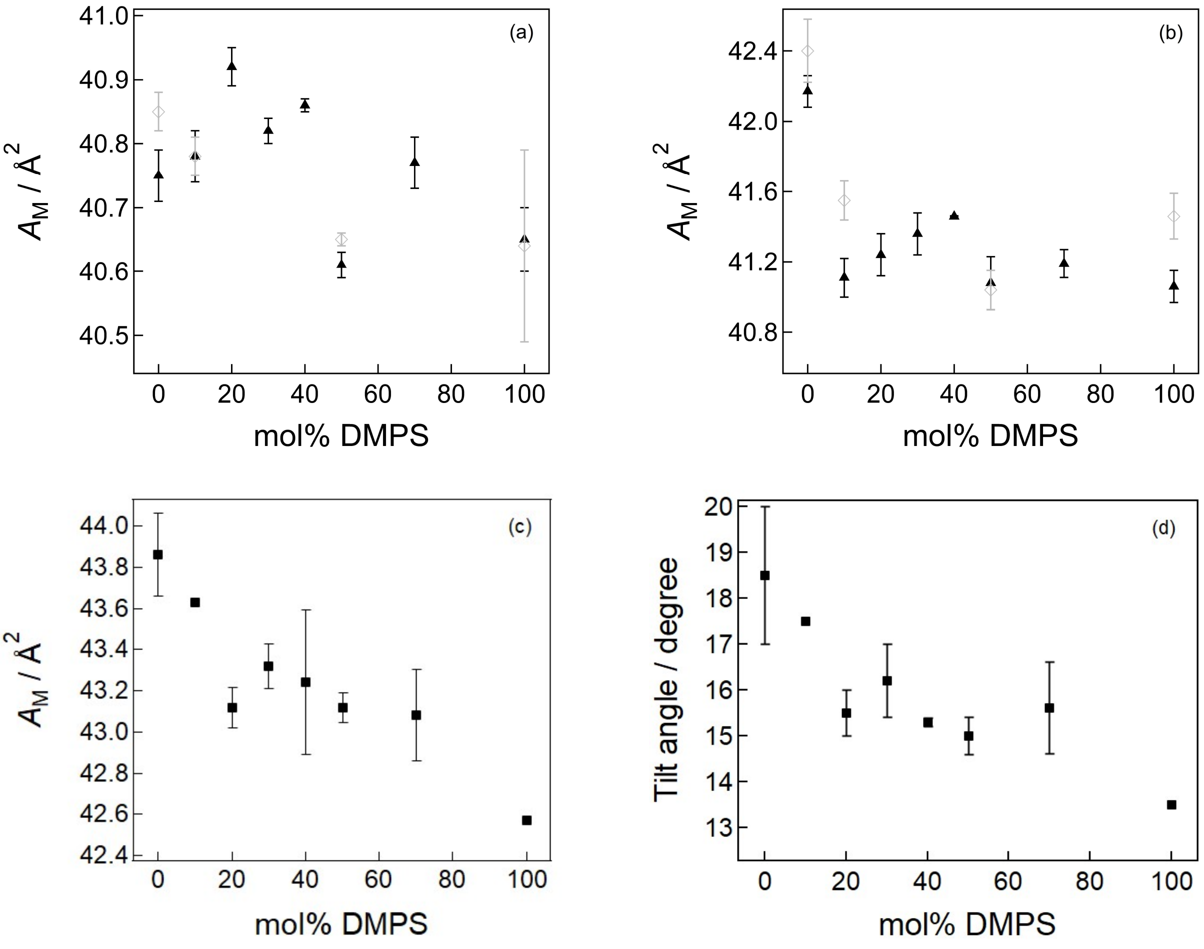

Figure S10. (a) Area per molecule derived from GIXD data at 40 mN m^{-1}: filled triangles h-lipids, open diamonds d-lipids. (b) Area per molecule derived from GIXD data at area A1 (38 Å$^{2)}$: filled triangles h-lipids, open diamonds d-lipids. (c) Area per molecule derived from GIXD data at area A2 (42 Å^2). (d) Corresponding tilt angle of the hydrocarbon chains from the surface normal at area A2.

6. Neutron Reflectivity and X-ray Reflectivity Data

Reflectivity data were fitted with RasCal [S9] using a two-slab model, with a headgroup slab including all of the polar part of the molecules, including the carbonyl carbon atoms, and a tailgroup slab including all carbon atoms in the chains but the carbonyl groups, i.e., 13 carbon atoms in each chain. X-ray reflectivity (XRR) and neutron reflectivity (NR) could not be co-fitted, even if only data for per-deuterated chains were used, so XRR and NR data were fitted separately. In the case of NR, only the data from deuterated lipids on D_2O and ACMW were co-fitted because some differences in the XRR data for deuterated and undeuterated lipids were observed at lower surface pressures and in the GIXD of some samples. The parameters varied in the model are the thickness, scattering length density and roughness of each slab. To reduce the number of parameters fitted and to avoid potential issues of negative content,[S10] the roughnesses of each interface were kept the same. The scattering length density

depends on the total scattering length and the molecular volume of the portion of the molecule contained within the slab:

$$\rho = \frac{\sum_i^n b_i}{V_\mathrm{m}}$$

Eq. S6

Table S9 gives the neutron scattering lengths and the X-ray scattering lengths (total electrons multiplied by 2.182 × 10^{-5} Å) used for fitting, and commonly used molecular volume parameters from the literature. The scattering length density of the headgroup slab depends also on the volume fraction of solvent in the slab so, if the headgroup volume is known, the solvent content may be calculated from Eq. S7:

$$\rho_\mathrm{solvhg} = \phi_\mathrm{w}\,\rho_\mathrm{w} + (1-\phi_\mathrm{w})\;\rho_\mathrm{dryhg}$$ Eq. S7

where ρ_solvhg is the fitted scattering length density (SLD) of the solvated headgroup, ϕ_w is the volume fraction of water, ρ_w is the scattering length density of water and ρ_dryhg is the scattering length density of a "dry" or unsolvated lipid headgroup. In the case of mixtures, ρ_dryhg is taken as an average value for the given composition and the equation may be rearranged to obtain ϕ_w from the fitted SLD.

Table S9. Reflectivity parameters

Slab	b / 10^{-3} Å (neutrons)	b / 10^{-3} Å (X-rays)	volume / Å^3	ρ / 10^{-6} Å^{-2} (neutrons)	ρ / 10^{-6} Å^{-2} (X-rays)
Undeuterated chains ($C_{26}H_{54}$)	–2.9110	5.9178	245 (S) [S11]<br>663 (L_c) [S10]	–4.513 (S)<br>–4.391 (L_c)	9.175 (S)<br>8.926 (L_c)
Deuterated chains ($C_{26}D_{54}$)	5.3303	5.9178	245 (S) [S11]<br>663 (L_c) [S10]	8.264 (S)<br>8.040 (L_c)	9.175 (S)<br>8.926 (L_c)
PE headgroup $C_7H_{12}NO_8P$	0.6257	3.9452	251 [S11]	2.493	1.572
PS headgroup $C_8H_{11}NO_{10}P$	0.8456	4.5370	244 [S12]<br>321 [S13]	3.466<br>2.634	1.859<br>1.414
PE headgroup / D_2O (if ammonium group hydrogen atoms exchanged) $C_7H_9D_3NO_8P$	0.9378	3.9452	251 [S11]	3.737	1.572
PS headgroup / D_2O (if ammonium group hydrogen atoms exchanged) $C_8H_8D_3NO_{10}P$	1.1158	4.5370	244 [S12]<br>321 [S13]<br>278 [S14]	4.745<br>3.607	1.859<br>1.414

Unfortunately, it can be difficult to obtain reliable "dry" headgroup volumes from literature; for example, values for PS of 321 Å^3 and 244 Å^3 have been used in neutron reflectivity papers, the former derived from a Gaussian calculation[S13] and the latter obtained from X-ray diffraction,[S12] and 278 Å^3 was used for analyzing small-angle scattering data in ref. S14. In addition, if molecules are not ideally mixed, the average molecular volume may not necessarily be easily obtained from the individual molecular volumes. Further, both PE and PS have labile hydrogen atoms in their ammonium groups, which may undergo exchange with deuterium in the subphase in neutron experiments. The deuterium exchange would be expected to be fast in solution but the degree of this deuterium exchange in

monolayers is unknown. Hence, for XRR, there are two unknown parameters and one dataset and for NR there are three unknown parameters and two datasets. To circumvent the problem, we can make use of the fact that the surface excess (and therefore area per molecule) must be the same for each slab in the model, as shown in Figure S11. The methods used in each case to extract solvation information from the fitted parameters are described below.

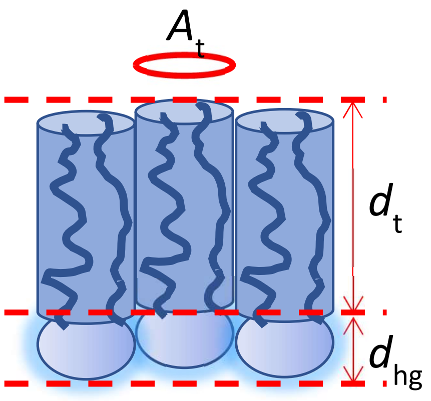

Figure S11. Cartoon depicting slabs used in reflectivity fitting and area per molecule in the tailgroup slab.

For our data, it is possible (in principle) to fit data with five parameters but they are heavily correlated. Fits were, therefore, made with the roughness fixed and other parameters free, using a series of values of roughness. Following this, the SLD of the hydrocarbon chains was also fixed for various values of tailgroup slab volume and fits obtained for each value. The results of each fit were examined and the best fit was chosen, in a manner similar to that described by Campbell et al.[S10] The best fit was selected according to whether the derived parameters and their combination were physically viable in preference, with the quality of the fit (χ^2) as a secondary consideration, as explained in each case below.

The thickness of the tailgroup slab was constrained with a maximum value of 17.9 Å (obtained from the Tanford equation[S15]) for monolayers with untilted chains, and 17.9cos(θ) Å for monolayers with tilted chains (using θ obtained from GIXD measurements at the same area per molecule or, for those samples where no diffraction was observed, from the measurement at the largest area per molecule where diffraction was observed). The thickness of the headgroup slab was constrained with a minimum value of 6 Å. The inclusion of solvent lowers the headgroup XRR SLD, so the maximum value was taken as that of the "dry" lipid headgroup, using a weighted average for mixtures (and the smallest literature volume (244 Å^3 [S12]) for PS). For NR, the same constraint was applied for the ACMW contrast. For D_2O, increasing solvation raises headgroup SLD and so the "dry" lipid headgroup SLD with no deuterium exchanged was taken as the minimum. The other boundaries varied according to the phase of the monolayer.

XRR

The fixed chain slab molecular volume was divided by tailgroup slab thickness to obtain an area per molecule. One criterion for a "good" fit is the closeness of this value to the area per molecule obtained from GIXD measurements or isotherm measurements. Two calculations were performed in parallel.

The first involved assuming the literature headgroup volumes in Table S10 to be correct (for PS each was tried) and rearranging equation S7 to obtain volume fraction of solvent:

$$\varphi_w = (\rho_{fitted} - \rho_{hg\_unsolv}) / (\rho_w - \rho_{hg\_unsolv}) \quad \text{Eq. S8}$$

Here, ρ_{hg_unsolv} is calculated from the scattering length and the literature volume.

The volume of the solvated headgroup was then calculated from the volume fraction of water and the "dry" headgroup volume. This value and the headgroup slab thickness were used to obtain area per molecule in the headgroup slab, which was then compared with the area per molecule of the tailgroup slab to determine plausibility of the model.

The second calculation assumed no knowledge of the headgroup volume but instead fixed the area per headgroup to the area per molecule of the tailgroup slab, A_t (obtained from the tailgroup slab volume and thickness). This area was multiplied by slab thickness d_{hg} to give the volume per molecule of the solvated headgroup, V_{hg_solv}:

$$V_{hg\_solv} = A_t \times d_{hg} \quad \text{Eq. S9}$$

The fitted scattering length density was multiplied by this volume to obtain the total scattering length of one (weighted average) lipid headgroup and n_w water molecules:

$$\rho_{fitted} \times V_{hg\_solv} = (b_{hg} + n_w b_w) \quad \text{Eq. S10}$$

From this value, n_w can be obtained because the scattering lengths are known. Once n_w is known, the "dry" lipid headgroup volume can be calculated, assuming one water molecule occupies 29.9 $Å^3$. The "dry" headgroup volume was compared with literature values to decide if it was physically reasonable.

The same calculation was performed in parallel using the areas per molecule obtained from GIXD data and the isotherms, in view of the fact that the error in the area per molecule from the fit may be larger.

DMPE headgroup volumes obtained via the second calculation were close to the literature value of 251 $Å^3$ but DMPS headgroup volumes varied. These were close to 244 $Å^3$ at lower surface pressures (higher areas per molecule) but increased in the solid phase to around 307 $Å^3$. Using the first calculation method, it was not possible to obtain fits with similar surface excess in each slab. A similar problem was encountered in fitting NR data. From these findings we conclude that the second type of calculation is necessary for some compositions, particularly those with higher DMPS content.

NR

As for XRR, the fixed chain slab molecular volume was divided by tailgroup slab thickness to obtain an area per molecule and a criterion for a "good" fit is the closeness of this value to the area per molecule obtained from GIXD measurements or isotherm measurements. As there were so many unknown parameters (headgroup volume, solvation and extent of deuterium exchange), rather than fixing "dry" headgroup SLD and fitting solvation, we fitted the headgroup slab SLDs for each contrast freely and selected fits where the results appeared compatible.

For the ACMW subphase, the SLD is zero and all scattering is from the lipid headgroup. The SLD of the solvated headgroup is then the scattering length of the headgroup divided by total solvated molecular volume and so the apparent solvated molecular volume can be calculated from:

$$V_{hg\_solv} = b_{hg} / \rho_{fitted} \quad \text{Eq. S11}$$

The area per molecule was then obtained from this volume and the slab thickness and compared with the value obtained from the tailgroup slab. This calculation was performed both for no hydrogen/deuterium exchange and for 8% of the labile hydrogen atoms exchanged.

If the extent of deuterium exchange is known, the SLD values from the two subphases can be used together to calculate volume fraction of water and volume of the dry headgroup. This calculation was carried out assuming no deuterium exchange and did not yield physically viable headgroup volumes. Instead, two parallel calculations were carried out as follows, the first assuming a known volume of the dry headgroup and the second aimed at calculating this value from the D_2O subphase contrast.

1a. Calculation with ACMW subphase contrast:

A value for "dry" headgroup volume was assumed from the literature and subtracted from the solvated headgroup volume obtained with Eq. 8 to obtain the volume of water associated with each lipid headgroup:

$$V_{hg\_solv} - V_{hg\_dry} = V_w \qquad \text{Eq. S12}$$

The volume fraction ϕ_w is then V_w / V_{hg_solv} and the number of water molecules per lipid, n_w, is $V_w / 29.9$.

1b. Calculation with D_2O subphase contrast

Eq. S7 can be rearranged to give Eq. S13:

$$\rho_{dryhg(D_2O)} = \frac{\rho_{fitted} - \varphi_w \rho_w}{1 - \varphi_w} \qquad \text{Eq. S13}$$

where ρ_{fitted} is the fitted SLD of the headgroup slab on D_2O and $\rho_{dryhg(D_2O)}$ is the SLD of the dry headgroup. The SLD of the D_2O is known (obtained from ensuring a good fit to the critical edge) and φ_w was obtained from the ACMW contrast calculation. Assuming the same headgroup volume as for the ACMW contrast, the scattering length can be obtained and used to estimate the extent of deuterium exchange.

2. Calculation with D_2O subphase contrast

This calculation is similar to that used for analyzing the XRR fits. The solvated headgroup volume is obtained from the tailgroup slab area per molecule and the headgroup slab thickness (Eq. S9).

The total scattering length of the headgroup slab is:

$$b_{solvhg(D_2O)} = b_{dryhg(D_2O)} + n_w b_{D_2O} \qquad \text{Eq. S14}$$

where b_{D_2O} is obtained from the fitted SLD of D_2O and the volume of a D_2O molecule. Rearranging for n_w gives:

$$n_w = \frac{b_{solvhg(D_2O)} - b_{dryhg(D_2O)}}{b_{D_2O}} = \frac{\rho_{solvhg(D_2O)} V_{solvhg(D_2O)} - b_{dryhg(D_2O)}}{b_{D_2O}} \qquad \text{Eq. S15}$$

n_w was calculated for different values of $b_{dryhg(D_2O)}$, one where no hydrogen/deuterium was exchanged, one for one atom exchanged, one for two atoms exchanged and one where three hydrogen atoms were exchanged.

Knowing n_w, the volume of water per lipid in the headgroup slab can be calculated, so, using Eq. S12, it is possible to calculate the volume of the dry lipid headgroup.

This dry lipid headgroup volume was compared with the literature value and that obtained from XRR fitting. It is also possible to use this value in the method outlined above for the ACMW contrast and verify that the number of water molecules obtained for the two contrasts is close.

Although this method is rather more cumbersome than co-fitting solvation for the two contrasts, it does allow for headgroup volume (or range of volumes) to be determined rather than assumed, which was necessary for the samples containing a high proportion of DMPS.

NR data with fits and corresponding SLD plots are provided in Figures S12–S16. XRR data with fits are given in Figures S17-S28. In each case, the solid lines are the fits and the shaded regions represent 95% confidence. Tables S10-S15 present the structural parameters derived from these fits. Figure S29 presents the total thickness at 47 mN/m as a function of composition and the number of water molecules per headgroup as a function of composition at each surface pressure and molecular area.

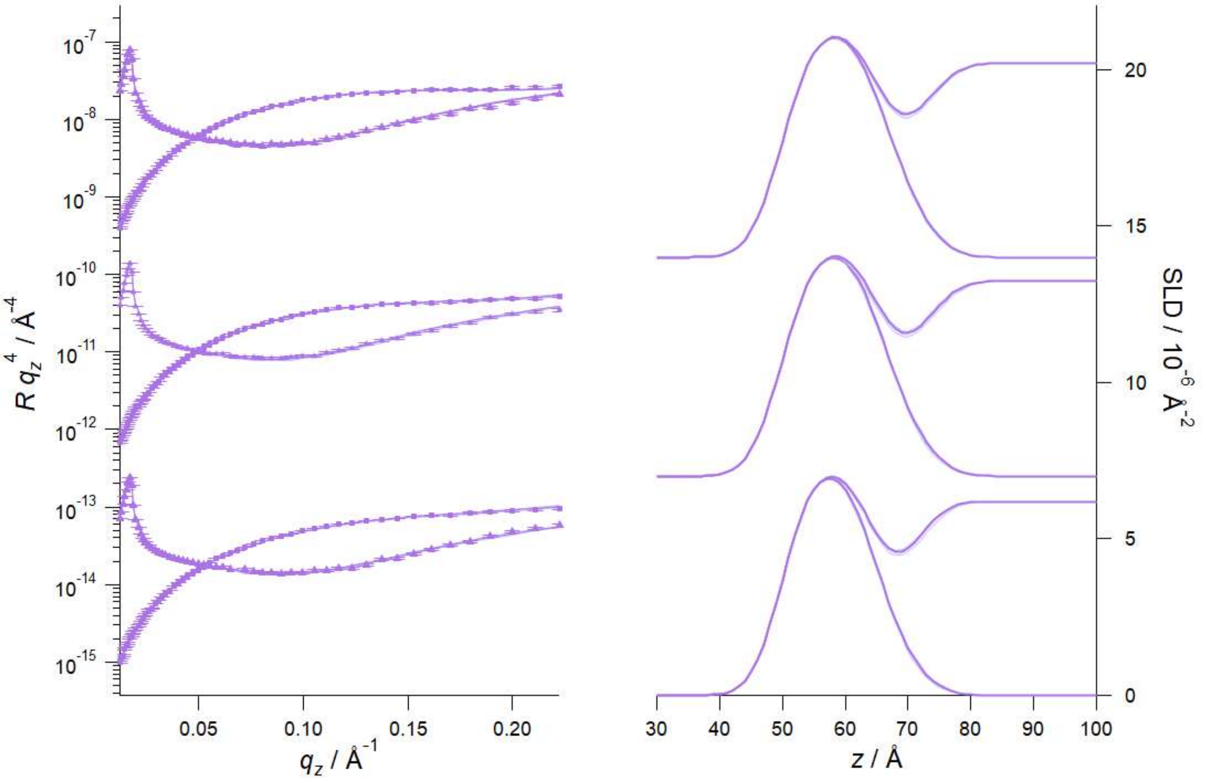

Figure S12. Left: NR data and fits for d-DMPE. Triangles d-lipid on D_2O, circles d-lipid on ACMW. Right: corresponding SLD plots. Data are offset by equal increments. Top to bottom: 40 mN m^{-1}, 38 Å^2, 46 Å^2.

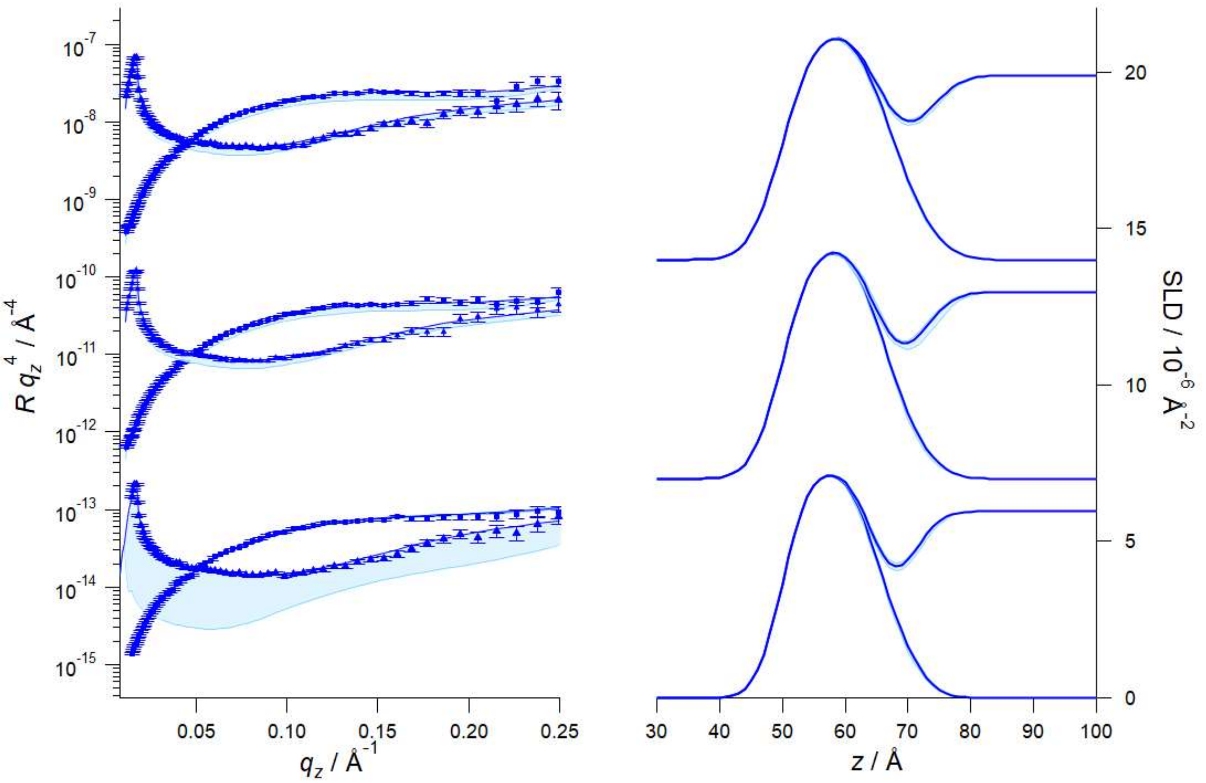

Figure S13. Left: NR data and fits for 9:1 d-DMPE:d-DMPS. Triangles d-lipid on D_2O, circles d-lipid on ACMW. Right: corresponding SLD plots. Data are offset by equal increments. Top to bottom: 40 mN m^{-1}, 38 Å^2, 46 Å^2.

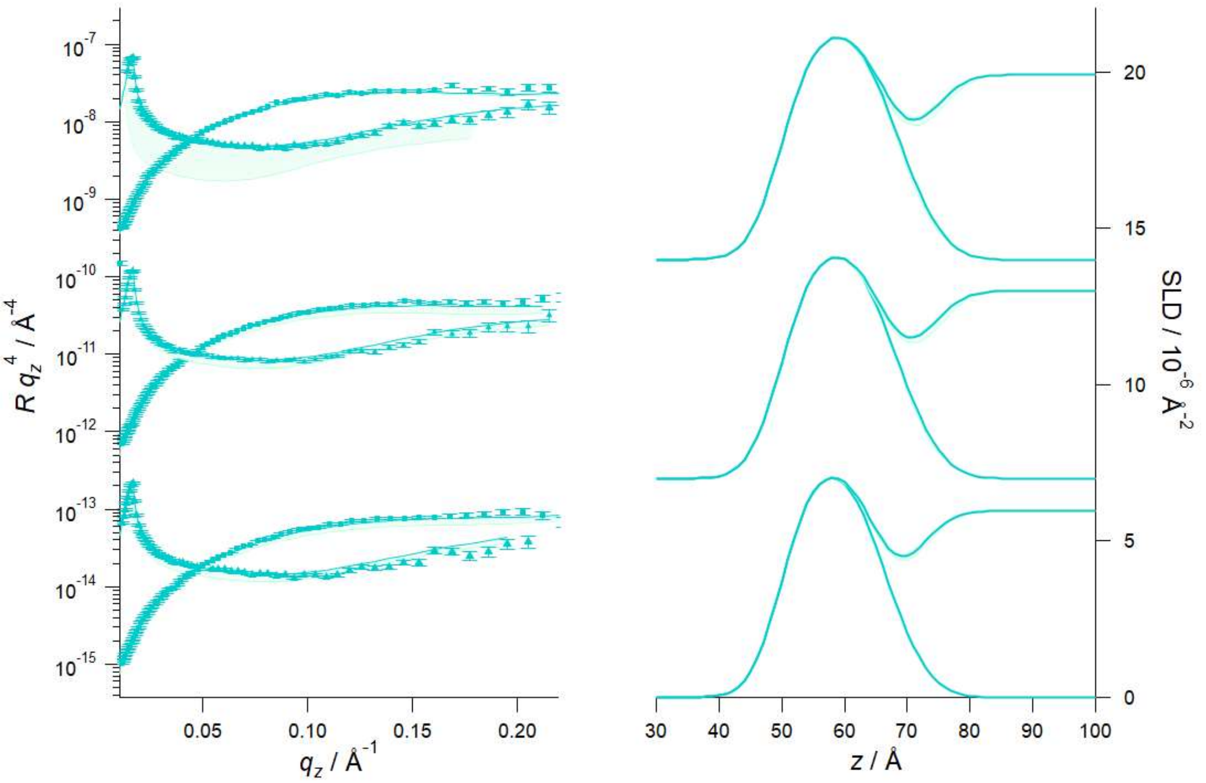

Figure S14. Left: NR data and fits for 7:3 d-DMPE:d-DMPS. Triangles d-lipid on D_2O, circles d-lipid on ACMW. Right: corresponding SLD plots. Data are offset by equal increments. Top to bottom: 40 mN m^{-1}, 38 Å^2, 46 Å^2.

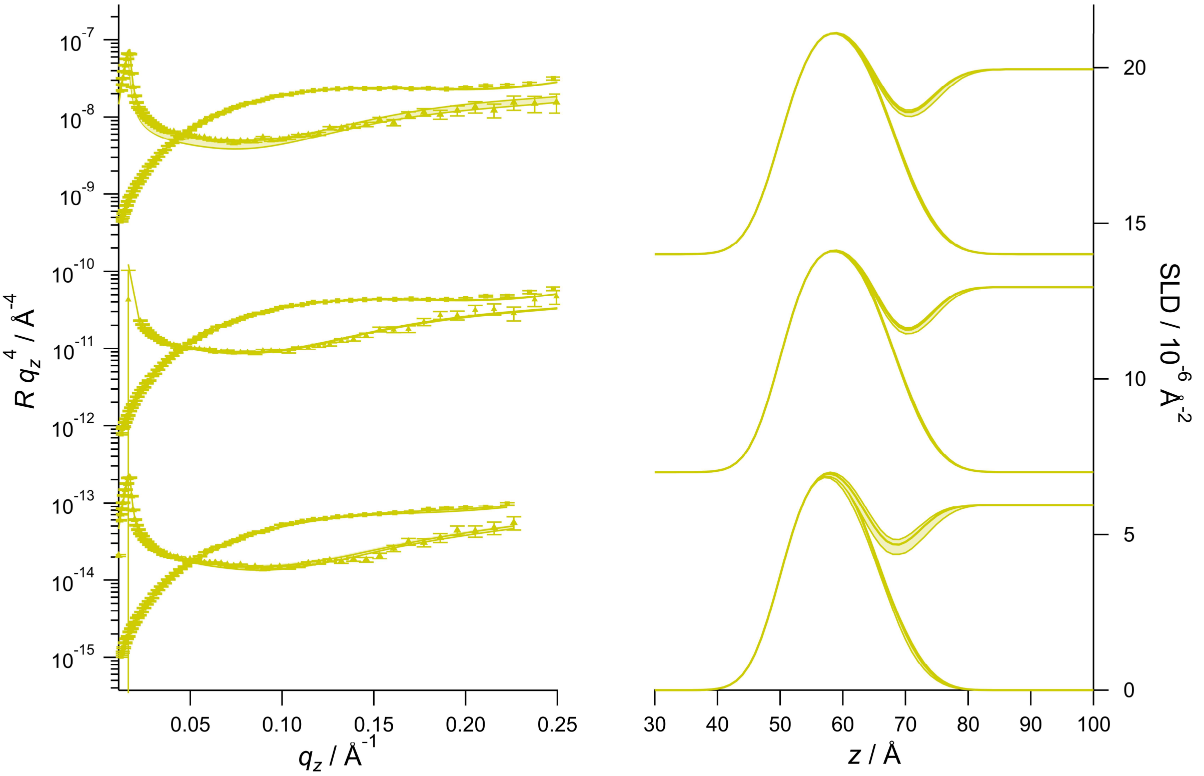

Figure S15. Left: NR data and fits for 1:1 d-DMPE:d-DMPS. Triangles d-lipid on D_2O, circles d-lipid on ACMW. Right: corresponding SLD plots. Data are offset by equal increments. Top to bottom: 40 mN m^{-1}, 38 Å^2, 46 Å^2.

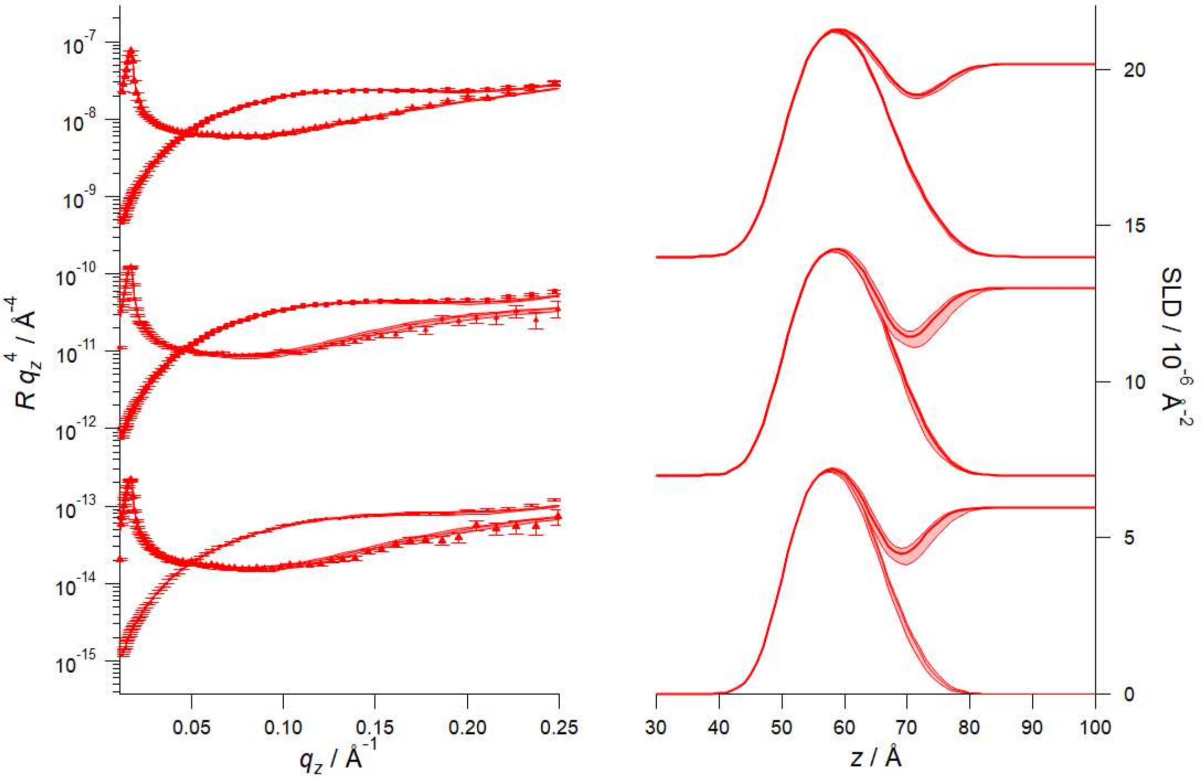

Figure S16. Left: NR data and fits for d-DMPS. Triangles d-lipid on D_2O, circles d-lipid on ACMW. Right: corresponding SLD plots. Data are offset by equal increments. Top to bottom: 40 mN m^{-1}, 38 Å^2, 46 Å^2.

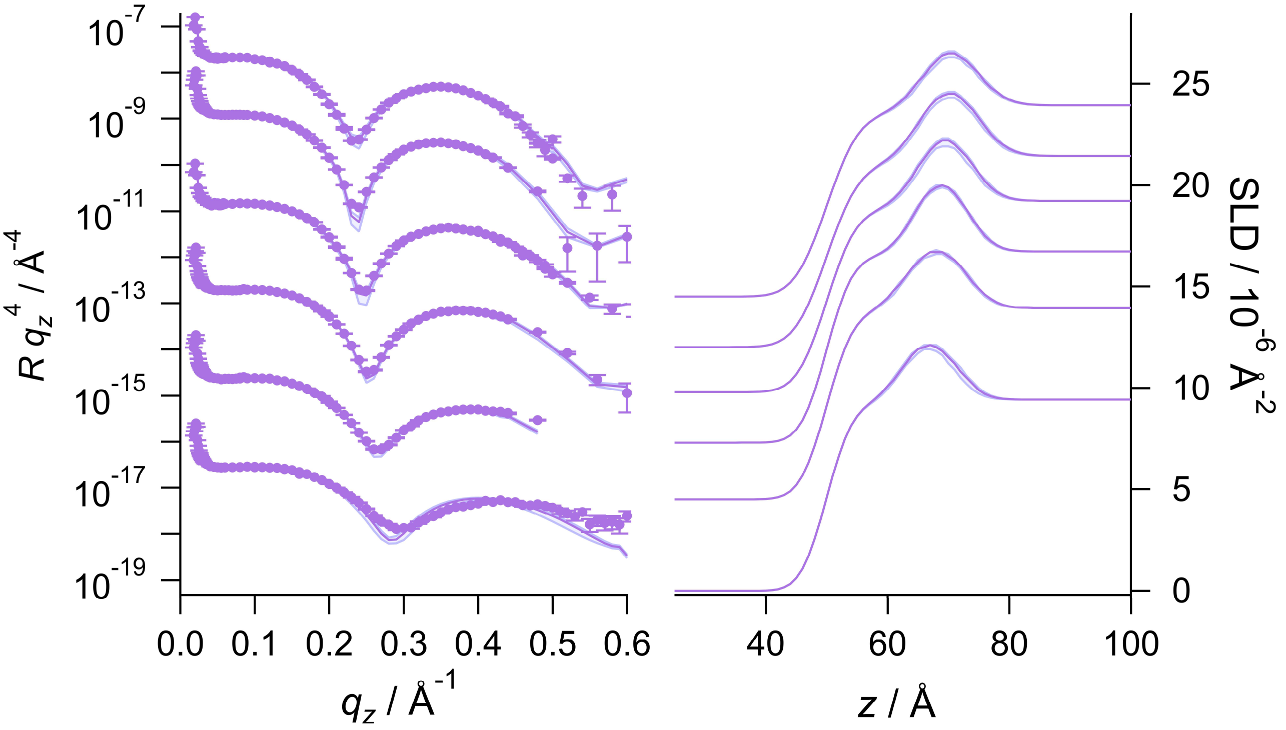

Figure S17. Left: XRR data and fits for h-DMPE. Right: Corresponding SLD plots. Data are offset by equal increments. Top to bottom: 47 mN m^{-1}, 40 mN m^{-1}, 38 Å^2, 42 Å^2, 44 Å^2, 46 Å^2.

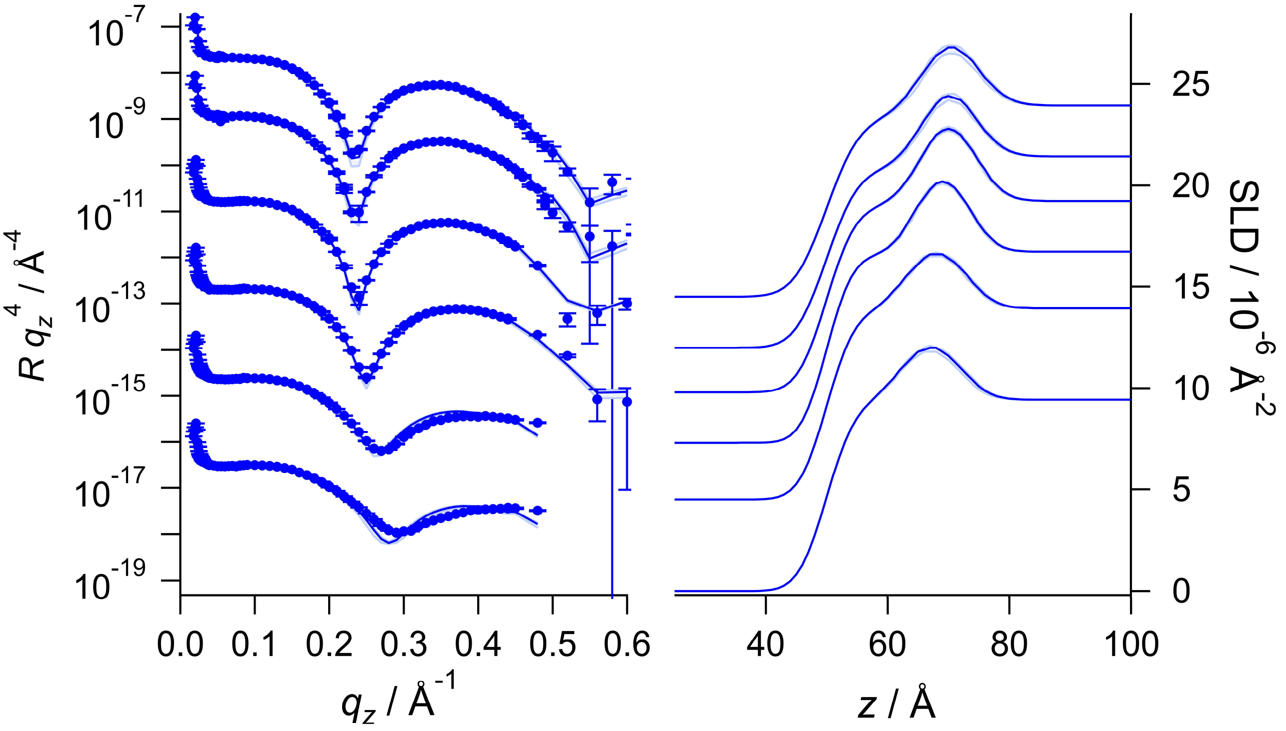

Figure S18. Left: XRR data and fits for 9:1 h-DMPE:h-DMPS. Right: Corresponding SLD plots. Data are offset by equal increments. Top to bottom: 47 mN m^{-1}, 40 mN m^{-1}, 38 Å^2, 42 Å^2, 44 Å^2, 46 Å^2.

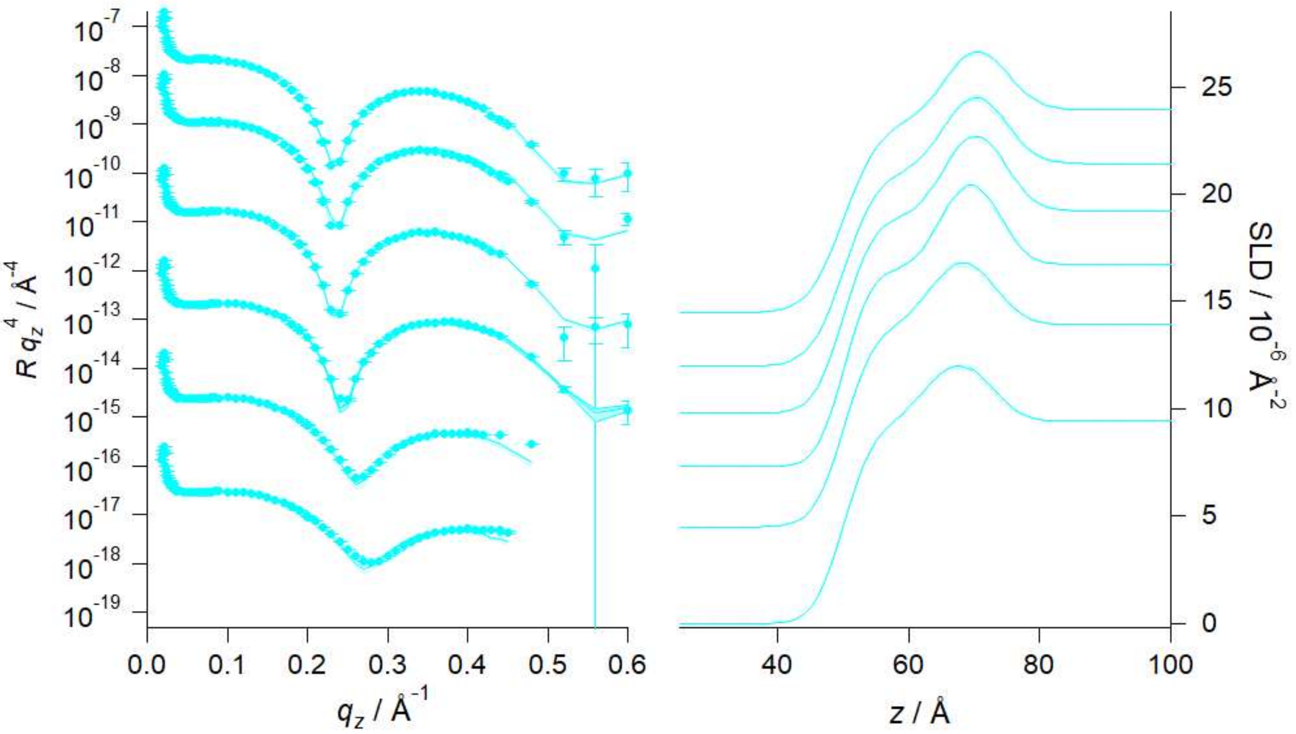

Figure S19. Left: XRR data and fits for 8:2 h-DMPE:h-DMPS. Right: Corresponding SLD plots. Data are offset by equal increments. Top to bottom: 47 mN m^{-1}, 40 mN m^{-1}, 38 Å^2, 42 Å^2, 44 Å^2, 46 Å^2.

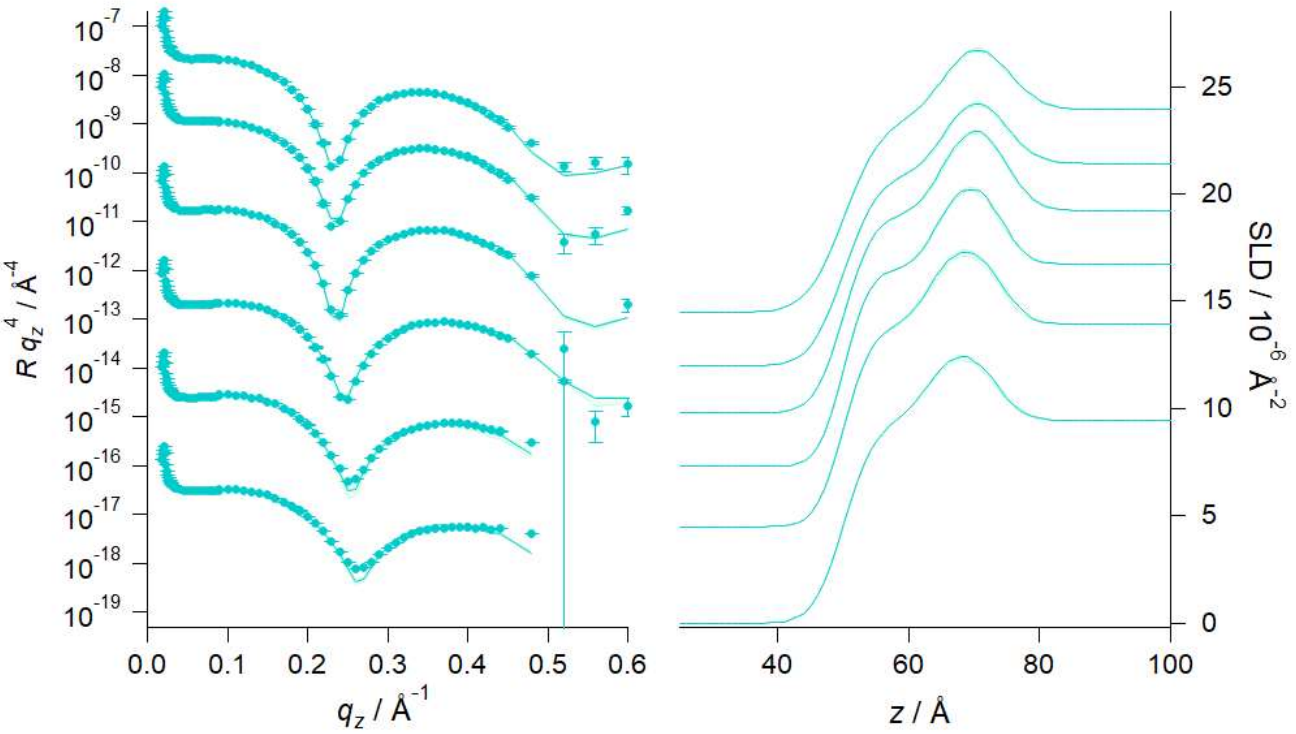

Figure S20. Left: XRR data and fits for 7:3 h-DMPE:h-DMPS. Right: Corresponding SLD plots. Data are offset by equal increments. Top to bottom: 47 mN m^{-1}, 40 mN m^{-1}, 38 Å^2, 42 Å^2, 44 Å^2, 46 Å^2.

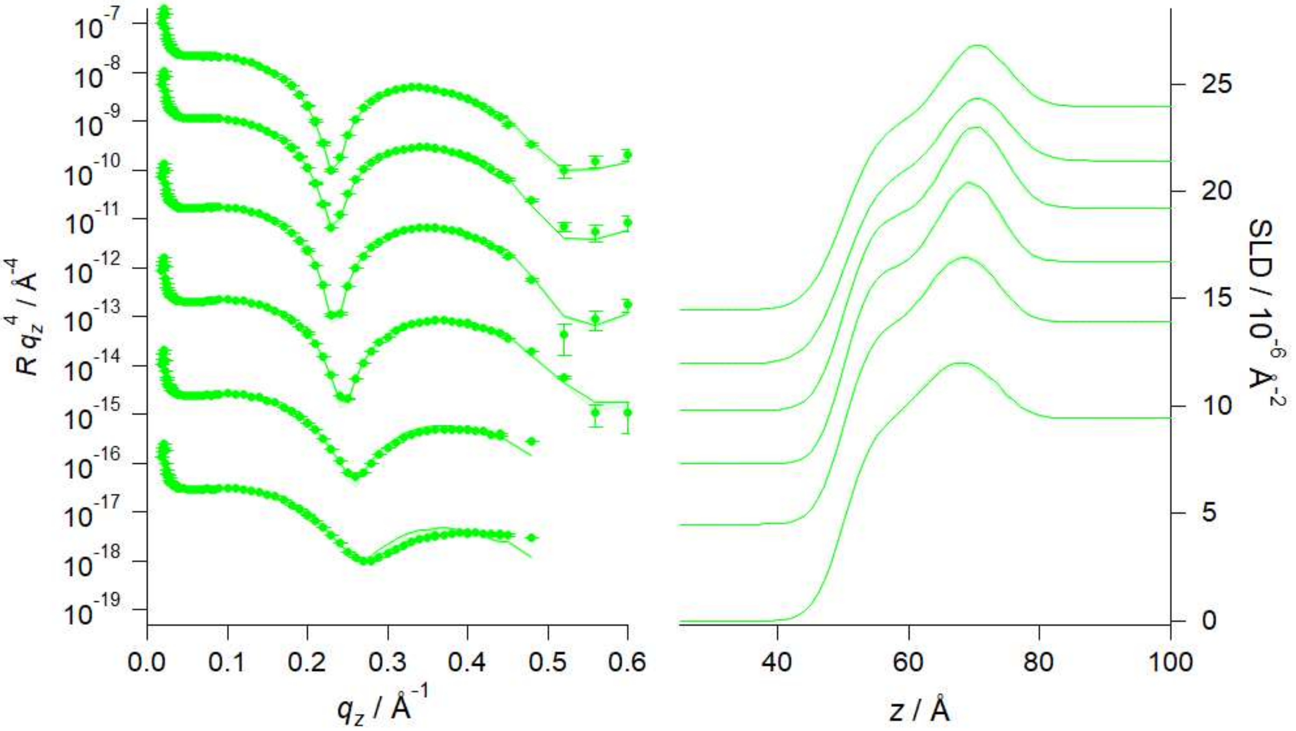

Figure S21. Left: XRR data and fits for 6:4 h-DMPE:h-DMPS. Right: Corresponding SLD plots. Data are offset by equal increments. Top to bottom: 47 mN m^{-1}, 40 mN m^{-1}, 38 Å^2, 42 Å^2, 44 Å^2, 46 Å^2.

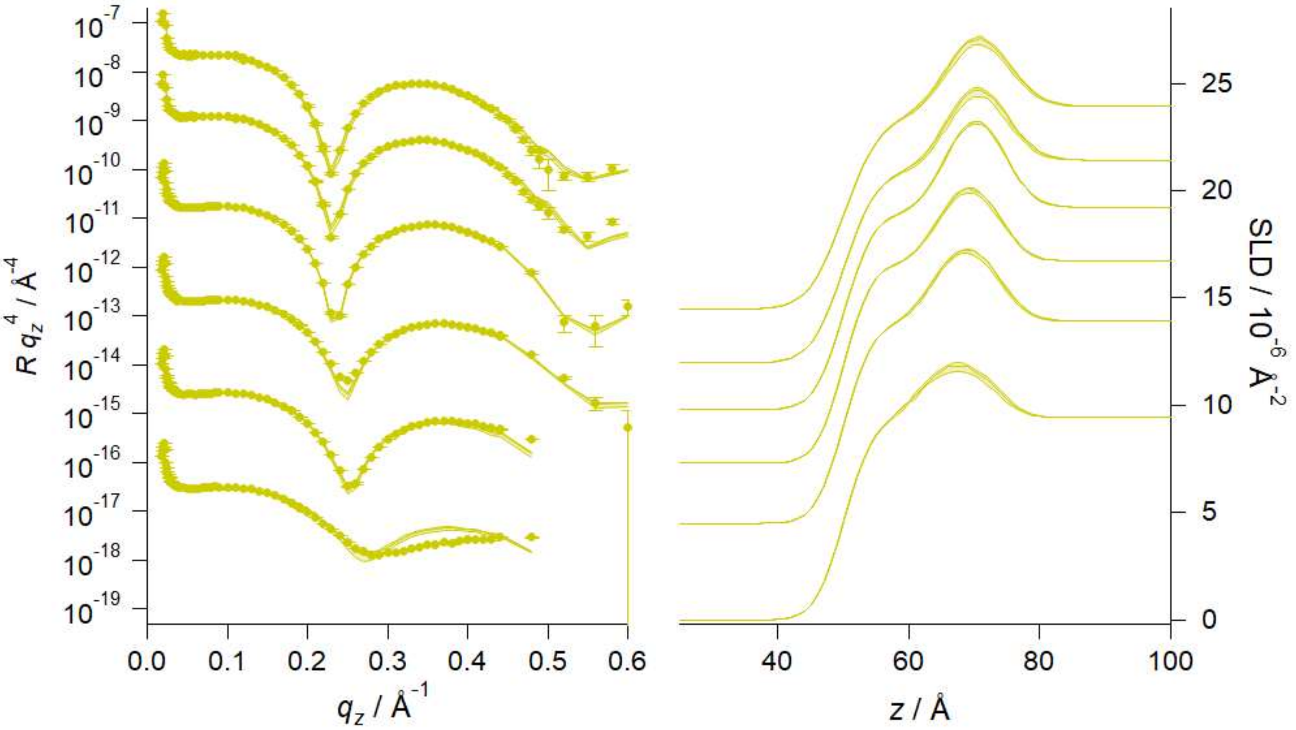

Figure S22. Left: XRR data and fits for 1:1 h-DMPE:h-DMPS. Right: Corresponding SLD plots. Data are offset by equal increments. Top to bottom: 47 mN m^{-1}, 40 mN m^{-1}, 38 Å^2, 42 Å^2, 44 Å^2, 46 Å^2.

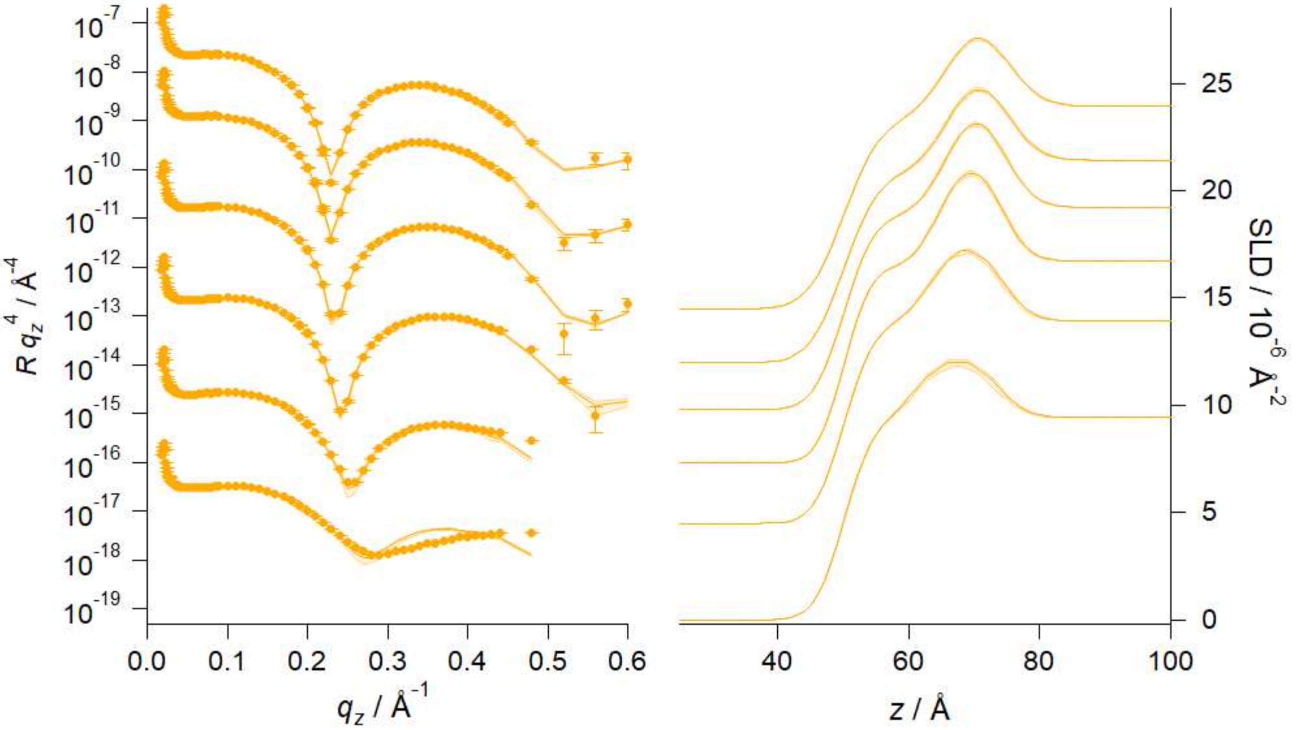

Figure S23. Left: XRR data and fits for 3:7 h-DMPE:h-DMPS. Right: Corresponding SLD plots. Data are offset by equal increments. Top to bottom: 47 mN m^{-1}, 40 mN m^{-1}, 38 Å^2, 42 Å^2, 44 Å^2, 46 Å^2.

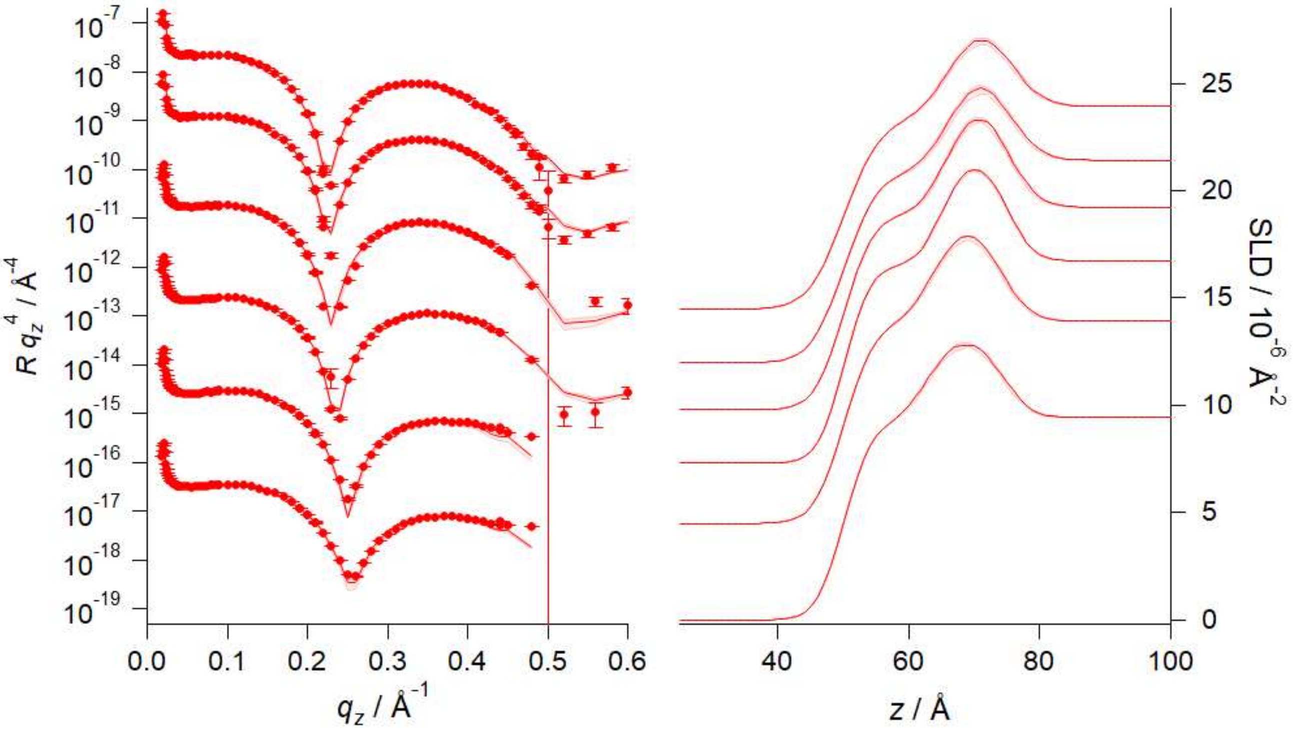

Figure S24. Left: XRR data and fits for h-DMPS. Right: Corresponding SLD plots. Data are offset by equal increments. Top to bottom: 47 mN m^{-1}, 40 mN m^{-1}, 38 Å^2, 42 Å^2, 44 Å^2, 46 Å^2.

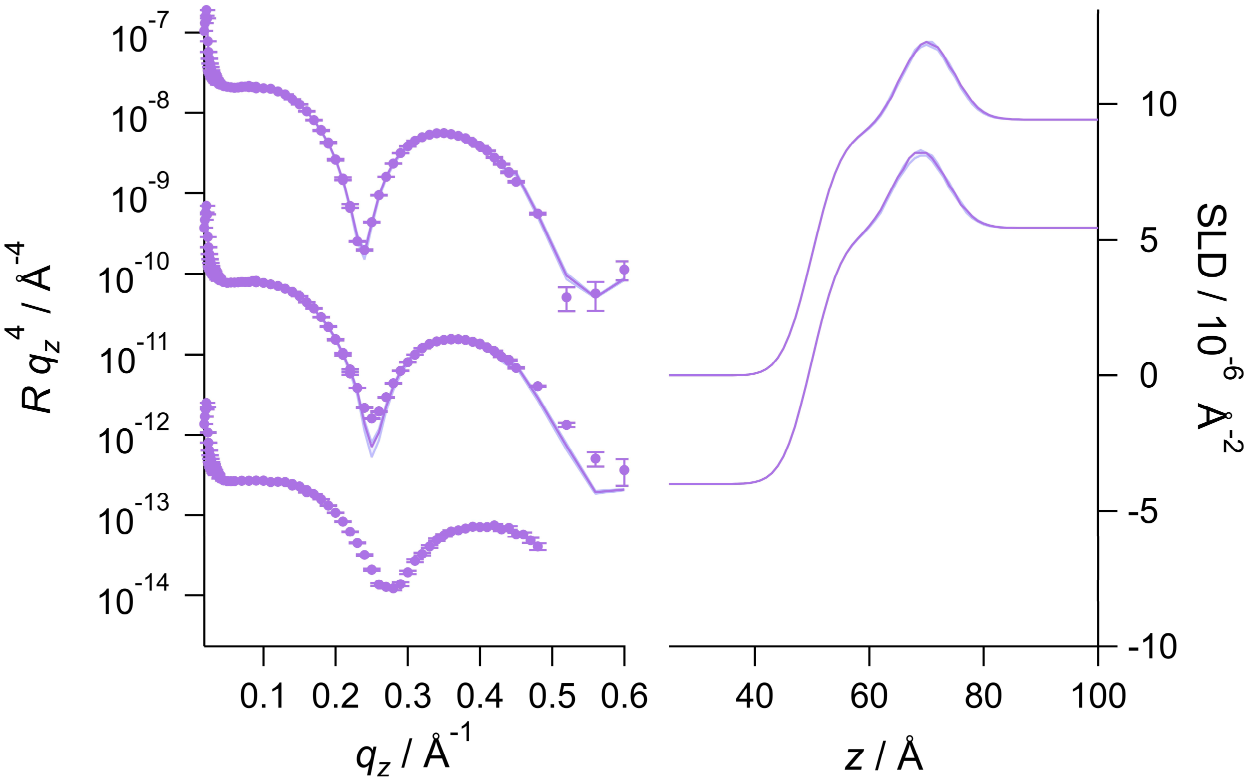

Figure S25. Left: XRR data and fits for d-DMPE. Right: Corresponding SLD plots. Data are offset by equal increments. Top to bottom: 40 mN m^{-1}, 38 Å^2.

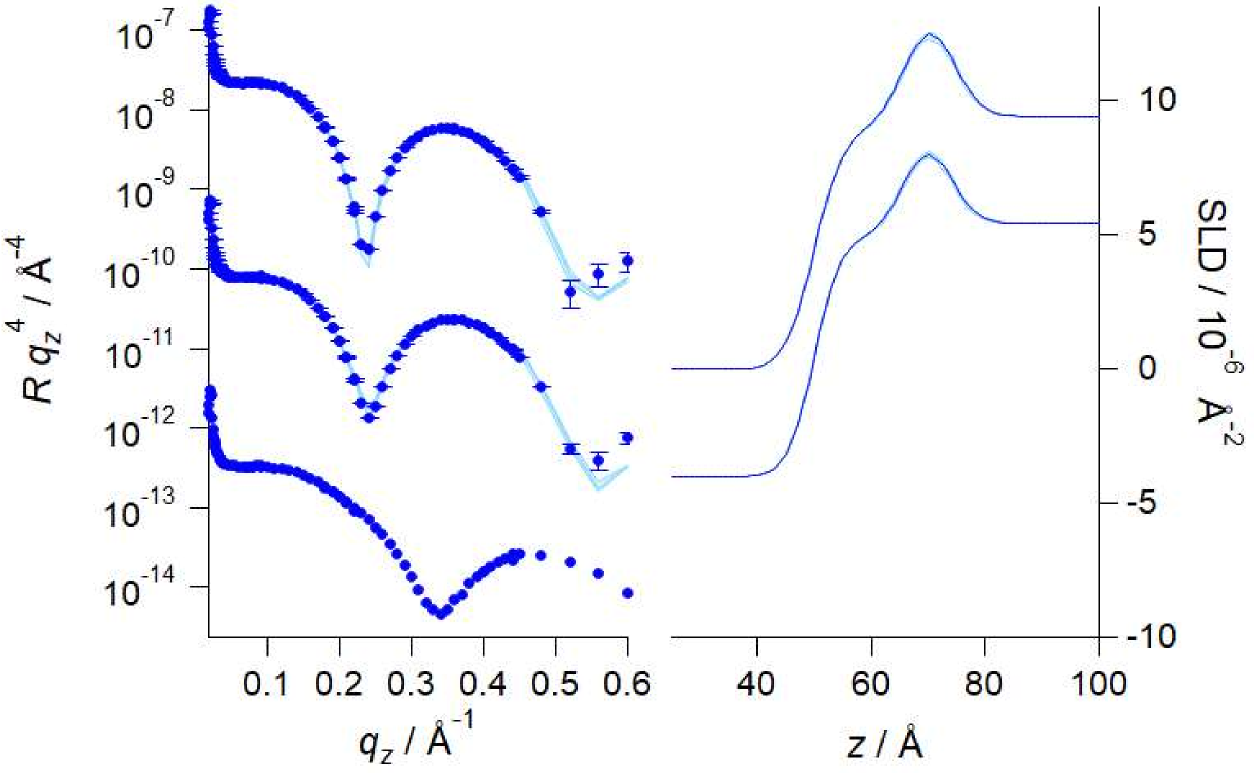

Figure S26. Left: XRR data and fits for 9:1 d-DMPE:d-DMPS. Right: Corresponding SLD plots. Data are offset by equal increments. Top to bottom: 40 mN m^{-1}, 38 Å^2, 46 Å^2.

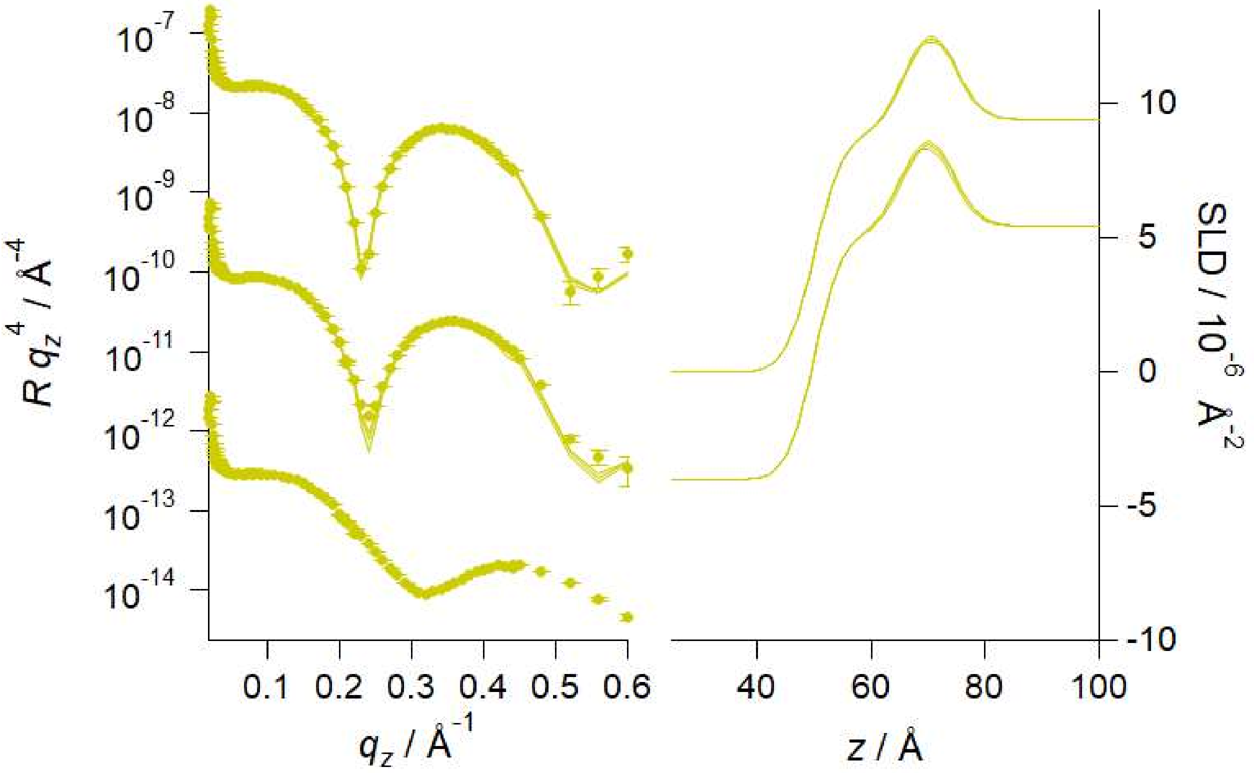

Figure S27. Left: XRR data and fits for 1:1 d-DMPE:d-DMPS. Right: Corresponding SLD plots. Data are offset by equal increments. Top to bottom: 40 mN m^{-1}, 38 Å^{2}.

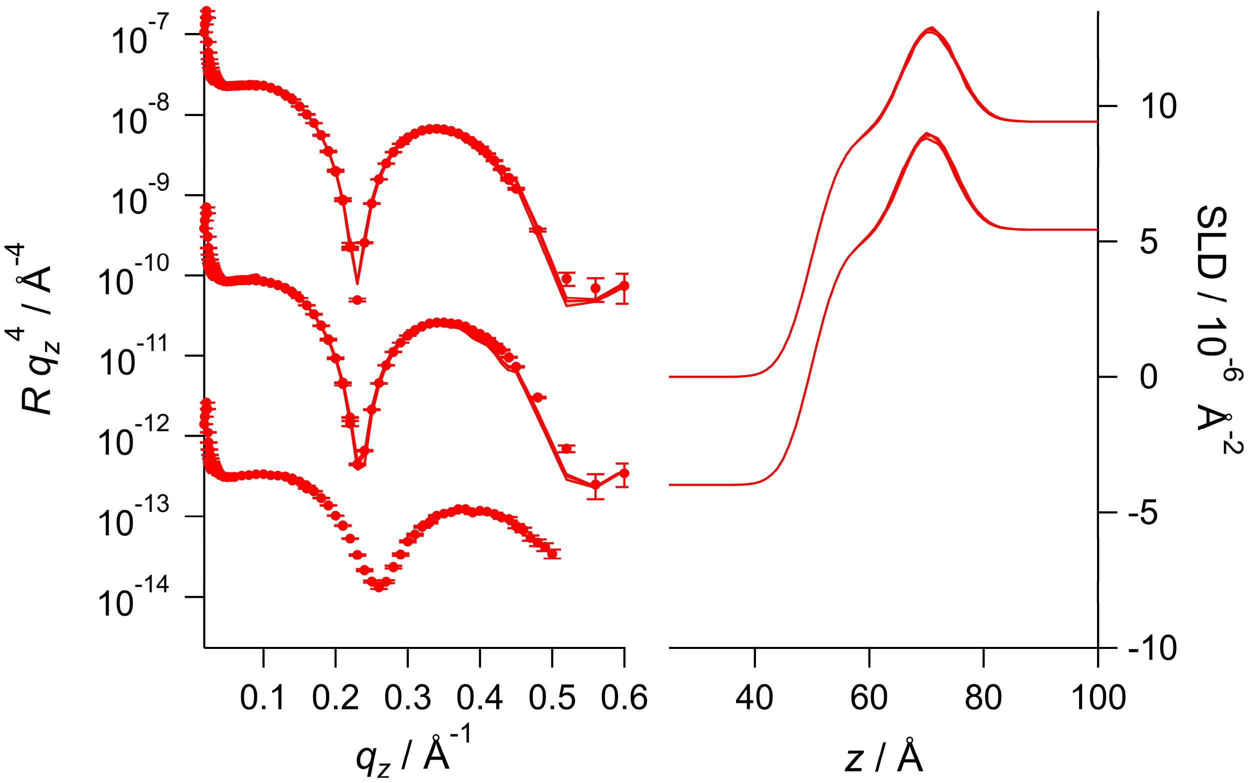

Figure S28. Left: XRR data and fits for d-DMPS. Right: Corresponding SLD plots. Data are offset by equal increments. Top to bottom: 40 mN m^{-1}, 38 Å^{2}.

Table S10. 47 mN m^{-1}

% DMPS	0	10	20	30	40	50	70	100
d_t / Å	16.6 (0.1)	16.7 (0.4)	16.6 (0.3)	16.5 (fixed)	16.6 (0.2)	16.7 (0.3)	16.6 (0.2)	16.9 (0.2)
d_{hg} / Å	6.7 (0.2)	7.0 (0.8)	7.2 (0.6)	7.5 (0.1)	7.1 (0.6)	7.5 (0.7)	7.6 (0.4)	7.7 (0.5)
A_M / Å^2	39.8 (0.3)	40.5 (1.0)	40.7 (0.7)	40.6 (0.6)	40.9 (0.7)	40.1 (0.9)	40.7 (0.6)	40.2 (0.8)
V_t / Å^3	660	675	675	670	680	670	675	680
V_{hg} / Å^3	275 (10)	271 (35)	282 (24)	279 (7)	283 (24)	282 (32)	286 (20)	311 (22)
n_w	0	0.38	0.4	0.81	0.32	0.59	0.78	0
σ / Å	4.2	4.3	4.35	4.5	4.4	4.35	4.4	4.35

Table S11. 40 mN m⁻¹

% DMPS	0	10	20	30	40	50	70	100
X-rays h-lipids:								
d_t / Å	16.1 (0.4)	16.5 (0.3)	16.1 (0.13)	16.4 (0.2)	16.4 (0.2)	16.5 (0.3)	16.5 (0.2)	16.8 (0.3)
d_{hg} / Å	7.7 (0.8)	6.9 (0.7)	7.9 (0.2)	7.4 (0.5)	7.7 (0.1)	7.8 (0.6)	7.8 (0.6)	7.8 (0.6)
A_M / Å^2	40.9 (1.0)	40.8 (0.9)	41.0 (0.4)	41.0 (0.7)	40.9 (0.6)	40.7 (0.8)	40.8 (0.7)	40.1 (0.8)
V_t / Å^3	660	675	660	675	670	670	675	675
V_{hg} / Å^3	252 (43)	271 (30)	267 (11)	288 (22)	283 (7)	282 (28)	283 (26)	307 (24)
n_w	2.15	0.41	1.86	0.53	1.12	1.10	1.17	0.16
σ / Å	4.2	4.1	4.1	4.1	4.3	4.0	4.1	4.1
X-rays d-lipids:								
d_t / Å	16.4 (0.2)	16.4 (0.3)				16.5 (0.2)		16.5 (0.2)
d_{hg} / Å	7.1 (0.4)	7.2 (0.7)				7.4 (0.5)		7.9 (0.4)
A_M / Å^2	40.5 (0.6)	40.50 (0.8)				40.5 (0.6)		40.5 (0.6)
V_t / Å^3	660	665				670		670
V_{hg} / Å^3	272 (18)	267 (31)				293 (22)		300 (20)
n_w	0.47	0.85				0.19		0.74
σ / Å	4.12	4.15				4.1		4.2
Neutrons:								
d_t / Å	15.6 (0.2)	16.1 (0.2)		16.8 (0.3)		16.3 (0.3)		16.2 (0.3)
d_{hg} / Å	6.5 (0.5)	6.2 (0.4)		6.3 (0.5)		6.2 (0.4)		8.3 (0.4)
A_M / Å^2	46.0 (0.6)	44.8 (0.7)		42.9 (0.9)		44.0 (0.8)		43.8 (0.8)
V_t / Å^3	720	720		720		715		710
V_{hg} / Å^3 *top: using tail A_M, bottom: using GIXD A_M. No. after slash for 1H/D exch.*	262 /282 (22)<br><br>244 /264 (22)	264 (21)<br><br>250 (20)		264 (23)<br><br>257 (22)		269 (17)<br><br>258 (16)		237 /254 (28)<br><br>230/247 (26)
n_w	1.23/0.55<br>0.71/0.03	0.45/0<br>0.09/0		0.19/0<br>0/0		0.15/0<br>0/0		4.17/3.61<br>3.54/2.97
n_w *(ACMW, assuming lit. vol.)*	0.3	0.6		0.3		0.4		3.0
σ / Å	4.2	4.2		4.3		4.3		4.1

Table S12. 38 Å²

% DMPS	0	10	20	30	40	50	70	100
X-rays h-lipids:								
d_t / Å	15.6 (0.3)	15.8 (0.2)	15.6 (0.1)	15.5 (0.1)	15.7 (0.1)	15.7 (0.1)	15.7 (0.1)	15.9 (0.3)
d_{hg} / Å	7.3 (0.7)	8.3 (0.4)	9.1 (0.2)	9.2 (0.2)	9.0 (0.1)	8.9 (0.2)	8.8 (0.2)	9.2 (0.6)
A_M / Å^2	42.3 (1.0)	40.9 (0.6)	41.7 (0.4)	41.7 (0.4)	41.4 (0.4)	41.4 (0.4)	41.6 (0.4)	41.2 (0.8)
V_t / Å^3	660	645	650	645	650	650	655	655
V_{hg} / Å^3	267 (32)	245 (24)	249 (12)	247 (12)	251 (13)	250 (11)	262 (13)	272 (34)
n_w	1.44	3.19	4.40	4.62	3.96	3.95	3.53	3.63
σ / Å	3.82	3.8	3.65	3.6	3.6	3.6	3.7	3.6
X-rays d-lipids:								
d_t / Å	15.3 (0.1)	15.9 (0.3)				15.6 (0.3)		16.1 (0.1)
d_{hg} / Å	7.3 (0.3)	7.6 (0.7)				8.4 (0.6)		8.2 (0.2)
A_M / Å^2	42.6 (0.5)	41.7 (0.9)				41.6 (0.9)		41.3 (0.4)
V_t / Å^3	650	665				650		665
V_{hg} / Å^3	268 (16)	294 (30)				290 (32)		293 (12)
n_w	1.45	0.76				1.95		1.47
σ / Å	4.1	3.8				3.9		4.1
Neutrons:								
d_t / Å	15.6 (0.3)	15.4 (0.4)		16.5 (0.3)		16.3 (0.2)		16.0 (0.8)
d_{hg} / Å	6.7 (0.6)	6.2 (0.4)		6.2 (0.3)		6.2 (0.4)		6.9 (0.7)
A_M / Å^2	46.3 (0.8)	46.1 (1.4)		43.7 (0.9)		44.5 (0.7)		44.7 (2.1)
V_t / Å^3	720	710		720		725		715
V_{hg} / Å^3 *top: using tail A_M, bottom: using GIXD A_M. No. after slash for 1H/D exch.*	263 /284 (34)<br><br>250 /270 (32)	270 (22)<br><br>254 (23)		267 (15)<br><br>259 (13)		259 /276 (16)<br><br>248 /266 (15)		289 /306 (38)<br><br>278 (34)
n_w	1.54/0.86<br>1.13/0.45	0.56/0<br>0.15/0		0.12/0<br>0/0		0.58/0<br>0.21/0		0.65/0.06<br>0.26/0
n_w *(ACMW, assuming lit. vol.)*	2.0	1.3		0.3		0.3		1.8
σ / Å	4.2	3.9		4.3		4.0		3.9

Table S13. 42 Å²

% DMPS	0	10	20	30	40	50	70	100
d_t / Å	14.5 (0.2)	14.6 (0.2)	14.8 (0.1)	14.4 (0.1)	14.5 (0.1)	14.4 (0.1)	14.6 (0.1)	15.1 (0.1)
d_{hg} / Å	8.6 (0.4)	8.9 (0.4)	9.3 (0.3)	9.8 (0.2)	9.7 (0.2)	9.5 (0.2)	9.7 (0.3)	9.9 (0.2)
A_M / Å^2	44.6 (0.7)	43.8 (0.6)	43.6 (0.5)	44.6 (0.5)	44.6 (0.5)	44.4 (0.4)	44.1 (0.5)	42.7 (0.4)
V_t / Å^3	645	640	645	645	645	640	645	645
V_{hg} / Å^3	250 (27)	248 (23)	240 (20)	251 (14)	247 (15)	270 (12)	243 (19)	261 (13)
n_w	4.45	4.67	5.45	6.30	6.26	5.14	6.23	5.36
σ / Å	3.4	3.4	3.35	3.15	3.3	3.4	3.3	3.25

Table S14. 44 Å²

% DMPS	0	10	20	30	40	50	70	100
d_t / Å	12.9 (0.2)	12.8 (0.1)	13.1 (0.2)	13.4 (0.2)	13.0 (0.2)	13.6 (0.2)	13.2 (0.2)	13.9 (0.2)
d_{hg} / Å	10.2 (0.4)	10.3 (0.3)	10.2 (0.5)	10.7 (0.5)	10.8 (0.2)	10.4 (0.4)	11.0 (0.5)	10.0 (0.5)
A_M / Å^2	49.6 (0.3)	49.8 (1.0)	48.9 (0.7)	47.9 (0.6)	49.1 (0.7)	47.1 (0.0)	48.3 (0.6)	46.1 (0.8)
V_t / Å^3	640	640	640	640	640	640	640	640
V_{hg} / Å^3	242 (31)	251 (23)	248 (40)	229 (41)	249 (19)	250 (34)	250 (40)	246 (37)
n_w	8.82	8.85	8.40	9.46	9.35	8.02	9.43	7.27
σ / Å	3.5	3.6	3.7	3.4	3.5	3.5	3.6	3.7

Table S15. 46 Å²

% DMPS	0	10	20	30	40	50	70	100
X-rays h-lipids:								
d_t / Å	12.1 (0.3)	12.0 (0.1)	12.3 (0.2)	13.0 (0.1)	11.8 (0.2)			12.8 (0.2)
d_{hg} / Å	9.4 (0.5)	10.0 (0.4)	10.5 (0.2)	10.1 (0.2)	12.3 (0.1)			11.7 (0.4)
A_M / Å²	53.3 (0.6)	53.2 (0.4)	51.9 (1.0)	49.1 (0.2)	54.4 (0.9)			50.5 (0.9)
V_t / Å³	645	640	640	640	640			645
V_{hg} / Å³	249 (45)	246 (31)	254 (41)	246 (15)	244 (36)			246 (33)
n_w	8.4	9.63	9.68	8.36	14.18			11.54
σ / Å	3.35	3.7	3.65	3.7	3.6			3.4
Neutrons:								
d_t / Å	14.3 (0.2)	14.4 (0.3)		15.2 (0.2)		14.3 (0.6)		14.8 (0.6)
d_{hg} / Å	6.7 (0.5)	6.2 (0.5)		6.1 (0.2)		6.4 (0.8)		6.9 (0.9)
A_M / Å²	50.7 (0.9)	50.6 (1.1)		47.4 (0.6)		50.7 (1.9)		48.8 (2.0)
V_t / Å³	725	730		720		725		720
V_{hg} / Å³ *top: using tail A_M, bottom: using isotherm A_M. No. after slash for 1H/D exch.*	261 /283 (34)<br>247 /268 (32)	270 /288 (32)<br>257 /273 (31)		271 /289 (13)<br>266 (18)		267 /285 (45)<br>254 /271 (43)		286 /303 (56)<br>278 /295 (49)
n_w	2.54/1.80 1.98/1.27	1.49/0.90 1.02/0.44		0.60/0.01 0.46/0		1.95/1.36 1.38/0.80		1.71/1.15 1.35/0.79
n_w *(ACMW, assuming lit. vol.)*	2.6	0.4		1.6		1.3		2.6
σ / Å	3.9	3.4		4.1		4.0		3.9

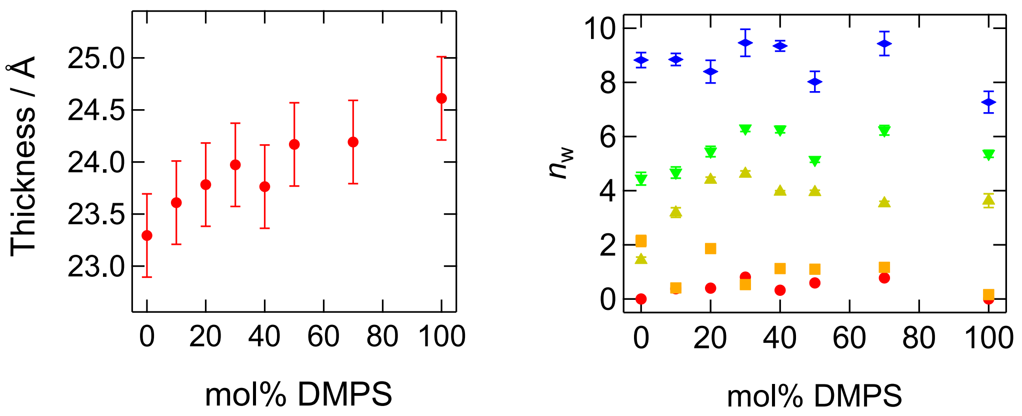

Figure S29. Left: Total monolayer thickness at 47 mN m^{-1}. Right: Number of water molecules per headgroup. Red circles 47 mN m^{-1}, orange squares 40 mN m^{-1}, yellow "up" triangles 38 Å^2, green "down" triangles 42 Å^2, blue diamonds 44 Å^2.
